# Electrostatic Potential at Nuclei vs. Atomic Charges as Descriptors of Hydrogen-Bond Basicity of Molecules

**DOI:** 10.3390/molecules31142438

**Published:** 2026-07-11

**Authors:** Ivan V. Atanasov, Diana Cheshmedzhieva, Sonia Ilieva, Boris Galabov, Henry F. Schaefer

**Affiliations:** 1 Faculty of Chemistry and Pharmacy, Sofia University “St. Kliment Ohridski”, 1164 Sofia, Bulgaria; i.atanas.031@gmail.com (I.V.A.); silieva@chem.uni-sofia.bg (S.I.); 2 Center for Computational Quantum Chemistry, University of Georgia, Athens, GA 30602, USA

**Keywords:** electrostatic potential at the nuclei, partial atomic charges, hydrogen-bond basicity, DFT

## Abstract

In this work, we comparatively assess the utility of electrostatic potential at nuclei (*V*_n_) and several frequently used atomic partial charge schemes for describing hydrogen-bond basicity. This is accomplished with a total of 114 examples across four classes of molecules: ketones, ethers, nitriles, and substituted pyridine derivatives. Density functional theory calculations at the CPCM//PBE0-D3/def2-TZVPP level were employed to evaluate reactivity descriptors for the most basic site in each molecule. The effectiveness of the descriptors was assessed through their correlation with experimental basicity data. Three widely used partial charge models—Hirshfeld, CM5, and NPA—were considered. Strong correlations between both *V*_n_ and partial charges with basicity were observed across all four molecular classes. Notably, the electrostatic potential at the nucleus (*V*_n_) consistently exhibits stronger correlations with the experimental pK_HB_ values than the partial charge models, highlighting its robustness as a quantitatively reliable descriptor of basicity across diverse organic systems. Both descriptors display inherent system dependence, and meaningful correlations with basicity are observed primarily within individual classes of compounds.

## 1. Introduction

Studies have revealed that the electrostatic potential at the nuclei (*V*_n_) provides a quantitative measure of the basicity and acidity of atomic sites, as reflected in hydrogen-bond-induced spectroscopic shifts, strong correlations with experimental acidity values, and chemical reactivities within series of related molecules [1,2,3,4,5,6,7,8]. This quantity is obtained directly from the molecular wave function without the introduction of additional approximations. Politzer and Truhlar [9] defined the electrostatic potential at the site of atom Y by the following expression:

(1) $$V_{Y}\equiv V\left(R_{Y}\right)=\sum_{A\neq Y}\frac{Z_{A}}{\left|R_{A}-R_{Y}\right|}-\int \frac{\rho \left(r^{\prime }\right)}{\left|r^{\prime }-R_{Y}\right|}dr^{\prime }$$

In Equation (1), *Z_A_* is the charge on nucleus A positioned at R_A_, and *ρ*(**r**) is the electron density function. The singular term for R_A_ = R_Y_ is excluded. Thus, Equation (1) accounts for the contributions from all other nuclei and from the total molecular electron density to the electrostatic potential at atom Y.

The recognition of the efficiency of the electrostatic potential at nuclei in quantifying reactivity of molecules in noncovalent interactions and chemical reactions [1,2,3,4,5,6,7,8] has led to numerous applications of *V*_n_ in characterizing properties and processes in chemistry and material science (see, e.g., refs. [10,11,12,13,14,15,16,17,18,19,20]). Suresh, Gadre et al. [17,18] illustrated the application of both special (global) minima of the molecular electrostatic potential (*V*_min_) and the electrostatic potential at nuclei (*V*_n_) as descriptors of chemical reactivity. Reviews covering the applications of the electrostatic potential at nuclei are also available [21,22,23]. In an earlier study, Murray et al. [24] established that *V*_min_ provides better correlations with experimentally derived hydrogen-bond donor and hydrogen-bond acceptor parameters than the minima of the surface electrostatic potential (*V*_S,min_).

Equation (1) represents a rigorous quantum-mechanical expression for the electrostatic potential at a nucleus and is, in this respect, superior to alternative local electronic descriptors such as atomic partial charges. By contrast, atomic charge definitions necessarily involve a number of approximations [25,26,27], resulting in different values for the same atomic site depending on the theoretical scheme employed. Nevertheless, atomic charges remain widely used for their clear physical interpretation and their demonstrated usefulness in describing local properties in molecules, polymers, and biomolecules [28,29,30]. By definition, the electrostatic potential at the nuclei is a quantity determined by contributions from both the immediate molecular environment and more distant fragments of the molecule. Despite this complexity, *V*_n_ values have been successfully applied in quantifying chemical reactivity [5,21].

In the present study, we compare the performance of electrostatic potential at nuclei and several types of atomic partial charges in describing the hydrogen-bond basicity for a series of molecules. The theoretical electronic parameters (*V_n_* and partial charges) are evaluated at the same level of density functional theory (PBE0-D3/def2-TZVPP). The correspondence between theoretical reactivity descriptors for atomic sites and experimental basicity values [31,32,33,34,35] is used to characterize the effectiveness of the electronic parameters studied. The present investigation examines trends of changes in basicity for a series of ketones, ethers, nitriles, and pyridine derivatives.

### Theoretical Methods

All quantum chemical computations were carried out within the framework of density functional theory at the PBE0-D3/def2-TZVPP level. The hybrid PBE0 exchange–correlation functional [36,37] has been shown to provide a reliable description of molecular electron density distributions [38]. The selection of the theoretical method in the present research is largely based on the results of a previous computational investigation [39] on the correspondence between theoretical atomic partial charges and experimental hydrogen-bond-induced frequency shifts for a series of nitrile derivatives. The results demonstrated [39] that the PBE0-D3 DFT method combined with the aug-cc-pVTZ basis set provides a quite reliable methodology for evaluating several different types of partial atomic charges for a series of nitrile derivatives. This research [39] assessed the performance of several density functional methods (B3LYP, ωB97X-D, PBE, PBE0, M06, M06-2X). It was also shown [39] that the Alrichs def2-TZVPP basis set [40] combined with the PBE0-D3 method provides correlation with the experimental frequency shifts in the same quality as when using the more computationally demanding PBE0-D3/aug-cc-pVTZ method. One of the aims of the present study was to employ an efficient theoretical method to derive information on molecular properties. The Alrichs def2-TZVPP basis set [40] is employed in the present study.

Dispersion interactions were accounted for consistently by applying the zero-damping D3 correction [41]. Because the experimental basicity data were determined in carbon tetrachloride solution [31,32,33,34,35], solvent effects were also modeled using the conductor-like polarizable continuum model (CPCM) [42,43].

All molecular geometries were fully optimized using tight self-consistent field convergence criteria together with an ultrafine integration grid (99, 590). Harmonic vibrational frequency computations were performed to verify that all optimized structures correspond to true minima on the potential energy surfaces, as confirmed by the absence of imaginary frequencies. All computations were conducted using the Gaussian 16 software package [44]. The Cartesian coordinates of all optimized structures are provided in the Supporting Information.

To characterize molecular basicity, three types of partial atomic charges were evaluated for the most basic reaction center of each molecule: Hirshfeld charges derived from the original stockholder partitioning scheme [45,46], Charge Model 5 (CM5) charges according to the procedure of Marenich et al. [47], and natural population analysis (NPA) charges based on the methodology developed by Weinhold and co-workers [48]. In parallel, the electrostatic potentials at the most basic atomic site of each molecule were also evaluated theoretically [9].

## 2. Results and Discussion

The theoretically derived partial atomic charges and electrostatic potential at the nucleus (*V_n_*) values for the principal basic reaction centers in the investigated systems were compared with the corresponding experimental hydrogen-bond basicity parameters, pK_HB_, across several series of organic molecules. The experimental pK_HB_ values were taken from the comprehensive studies of Laurence and co-workers [31,32,33,34,35]. These basicity parameters were obtained from measurements of the temperature dependence of the equilibrium constants for hydrogen bonding between 4-fluorophenol, as the proton-donating species, and the corresponding bases in carbon tetrachloride solution. Details of the experimental procedure for evaluating the pK_HB_ values are provided in ref. [34].

The degree of correspondence between the theoretical descriptors (*V_n_* and partial atomic charges) and the experimental pK_HB_ values was used as the principal criterion for evaluating the suitability of these quantities as descriptors of molecular basicity. In the following sections, the results are discussed for representative series of ketones, ethers, nitriles, and substituted pyridine derivatives.

### 2.1. Ketones

Table 1 summarizes the experimentally determined [31] hydrogen-bond basicity parameters (pK_HB_) together with the calculated Hirshfeld, CM5, and NPA atomic charges and the electrostatic potential at the nucleus [*V*_n_(O)] for the carbonyl oxygen atom in the ketone series. The set of compounds encompasses a broad range of structural and electronic environments, including open-chain and cyclic aliphatic ketones, substituted acetophenones, and benzophenone. As such, this series provides a suitable framework for evaluating the sensitivity of the theoretical descriptors to diverse factors affecting carbonyl basicity. These factors include inductive effects arising from strongly electron-withdrawing substituents in the α-position (e.g., CH_2_Cl and CF_3_), ring-strain effects in small cyclic ketones, and π-conjugative interactions involving one or two aromatic rings.

**Figure 1:**
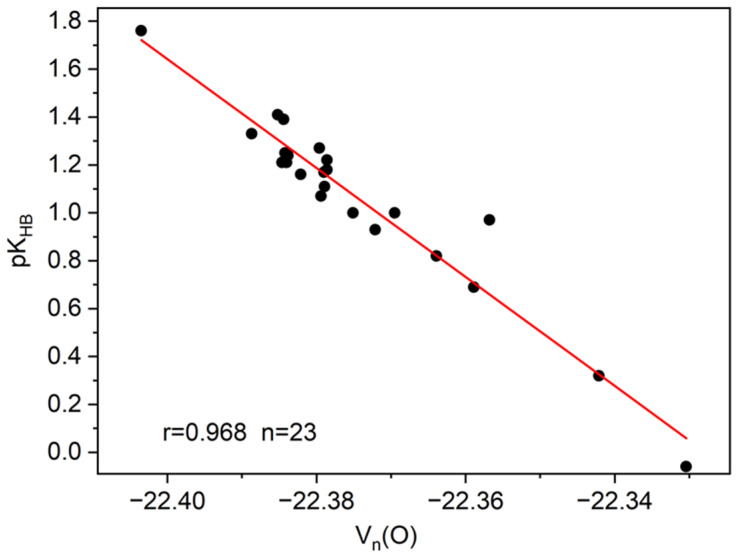
Linear correlation between the experimental hydrogen-bond basicity parameter pK_HB_ and the calculated electrostatic potential at the nucleus, *V*_n_(O), for the carbonyl oxygen atom in the ketone series.

As evident from the results in Table 1, all three charge models reproduce the experimental trends in basicity remarkably well. Linear correlations between the oxygen partial charge, *q*_O_, and pK_HB_ are observed throughout the series, with correlation coefficients of *r* = 0.945, 0.956, and 0.955 for Hirshfeld, CM5, and NPA charges, respectively. Most notably, the *V*_n_ descriptor yields the highest correlation, with *r* = 0.968 (*R*^2^ = 0.937), exceeding the performance of the three partial charge models. The corresponding linear relationship between pK_HB_ and *V*_n_(O) is shown in Figure 1. The superior performance of *V*_n_(O) suggests that the electrostatic potential at the nucleus provides a particularly sensitive measure of carbonyl basicity in ketones. The observed correlation implies that the experimentally measured hydrogen-bond basicity is influenced not only by the local electron population at the oxygen atom but also by substituent-induced electronic perturbations at the C=O oxygen transmitted through the molecular framework. Through-space intramolecular electrostatic contributions also affect, though less significantly [Equation (1)], the *V*_n_(O) values. At the same time, the close correspondence between the charge-based descriptors and *V*_n_(O) indicates that changes in local electron density at the carbonyl oxygen remain the dominant factor governing the basicity trend within this series.

### 2.2. Ethers

The results for the series of 29 ethers investigated are summarized in Table 2. The experimental hydrogen-bond basicities are taken from the study of Besseau et al. [32] This set encompasses a broad range of structural variations, including open-chain aliphatic ethers, strained cyclic ethers (trimethylene oxide, epichlorohydrin, 1,3-dioxolane, and furan), unsaturated ethers containing a vinyl group adjacent to the oxygen basic center, aliphatic ethers bearing bulky alkyl substituents (isopropyl and *tert*-butyl groups), as well as two crown ethers. Accordingly, the series provides a diverse representation of structural factors that may influence the electronic properties and hydrogen-bond basicity of the ether oxygen atom.

In contrast to the ketone series, a priori strong linear correlations between theoretically derived electronic descriptors and experimental basicity values are not necessarily expected for ethers. In this class of compounds, factors other than local electron density may significantly affect hydrogen-bond formation with the 4-fluorophenol proton donor. These include steric hindrance around the oxygen basic center, small-ring strain effects, and the presence of nearby polar groups capable of exerting through-space electrostatic influences. Such effects are particularly relevant for crown ethers, where the electrostatic environment at a given oxygen atom may be substantially perturbed by neighboring oxygen atoms within the cyclic framework. For this reason, compounds expected to exhibit particularly strong short-range perturbations—namely, sterically hindered ethers such as diisopropyl ether and di-*tert*-butyl ether, as well as the crown ethers—were excluded from the correlation analysis.

As is immediately evident from Table 2, none of the partial charge models reproduces the experimental hydrogen-bond basicity trends satisfactorily within the ether series. The correlation coefficients obtained for Hirshfeld, CM5, and NPA charges are *r* = 0.751, 0.709, and 0.728, respectively, indicating only modest relationships between the local oxygen charge and pK_HB_. These comparatively weak correlations likely reflect the substantial structural heterogeneity of the series and the increased importance of nonlocal effects governing hydrogen-bond complex formation.

**Figure 2:**
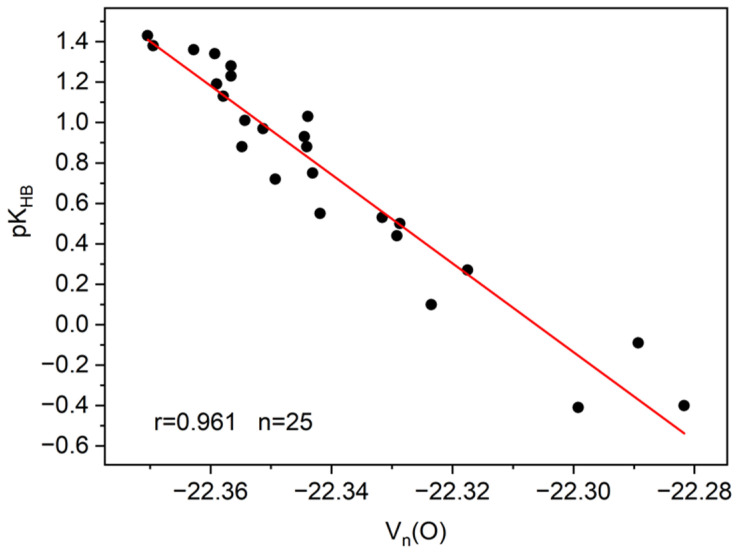
Linear correlation between the experimental hydrogen-bond basicity parameter pK_HB_ and the calculated electrostatic potential at the nucleus, *V*_n_(O), for the oxygen atom in the ether series.

By contrast, the electrostatic potential at the oxygen nucleus follows the experimental basicity trend remarkably well (Figure 2), yielding a substantially improved correlation coefficient of *r* = 0.961 (*R*^2^ = 0.923). This result is particularly noteworthy, given the pronounced structural diversity of the ethers investigated. The strong performance of the *V*_n_ descriptor suggests that it captures not only local electron-density variations at the oxygen atom, but also longer-range structural and electrostatic effects that are evidently important in this series. In this respect, the *V*_n_ appears to be superior to conventional partial atomic charge as a descriptor of molecular basicity for structurally diverse compounds within a given functional class. These findings are fully consistent with the results obtained for the ketone series and further support the use of the electrostatic potential at the nucleus as a robust theoretical descriptor of hydrogen-bond basicity.

### 2.3. Nitriles

Nitriles constitute a particularly suitable series for evaluating electronic effects at the C≡N nitrogen atom, as this reaction center is well separated from the rest of the molecular framework. Consequently, structural variations across the series do not occur in the immediate vicinity of the cyano group. As noted previously, spectroscopic studies [33] have demonstrated that steric effects do not significantly influence hydrogen-bond formation between 4-fluorophenol and nitrile bases. Strong correlations between computed partial charges and the experimentally observed Δν(OH)_exp_ shifts were established in an earlier study [39]. In particular, the hydrogen-bond-induced shifts in the O–H stretching frequency of 4-fluorophenol upon complexation with nitriles were found to correlate closely with Hirshfeld (*r* = 0.972) and CM5 (*r* = 0.967) partial charges on the nitrile nitrogen atom. (Table 3).

**Figure 3:**
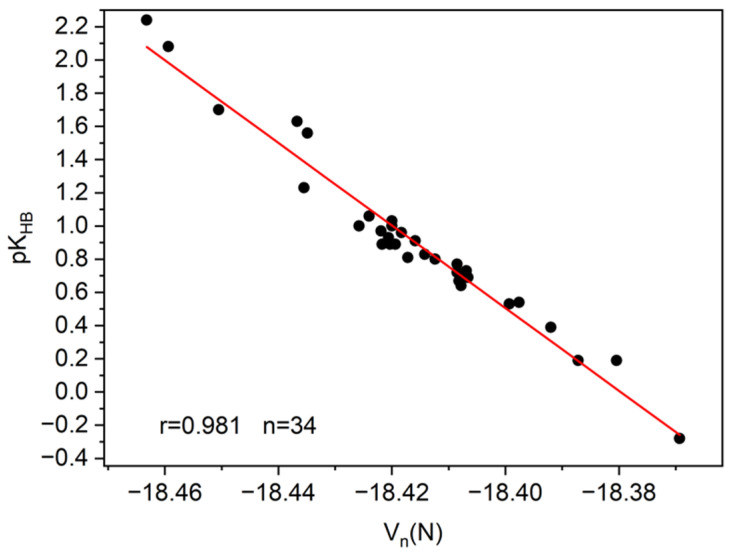
Plot of the relationship between the experimental hydrogen-bond basicity parameter pK_HB_ and the calculated electrostatic potential at the nucleus, *V*_n_(N), for the C≡N nitrogen atom in the nitrile series.

The present computations, performed at the PBE0-D3/def2-TZVPP level of theory, yield very similar trends. Partial charges derived using the NPA scheme exhibit a somewhat weaker correlation with pK_HB_ values. In contrast, the highest correlation (*r* = 0.981, *R*^2^ = 0.963) is again obtained using the electrostatic potential at the nucleus [*V*_n_(N)] as the descriptor of basicity. Figure 3 illustrates this relationship. This observation is consistent with the trends established for the ketone and ether series and further supports the reliability of electrostatic potential at the nucleus (*V*_n_) values as quantitative descriptors of the reactivity of basic centers in series of related molecules.

Although the electrostatic potential at a nucleus formally includes contributions from both local and distant regions of the molecule [Equation (1)], the present results indicate that *V*_n_(N) effectively captures basicity trends. This behavior can be rationalized by the inverse distance dependence of the contributing terms in Equation (1), which emphasizes local electronic and structural effects while attenuating contributions from more remote substituents.

### 2.4. Substituted Pyridines

The series of mono- and disubstituted pyridine derivatives at the 3-, 4-, and 5-positions provides a suitable framework for assessing the reliability of partial atomic charges and electrostatic potential at the nucleus [*V*_n_(N)] in capturing how structural variations within the heteroaromatic ring are reflected in these theoretical descriptors. In particular, this series enables evaluation of how effectively these parameters describe the influence of remote substituents on the basic reaction center.

Experimental hydrogen-bond basicity parameters (pK_HB_) [34], together with the computed descriptors for the pyridine nitrogen atom, are summarized in Table 4. Hirshfeld and CM5 partial charges reproduce the observed basicity trends with high fidelity, yielding correlation coefficients of *r* = 0.976 and 0.975, respectively. These results are notable in view of both the experimental uncertainties associated with basicity measurements and the approximations inherent in defining atomic charges. NPA charges provide a weaker, though still qualitatively correct, correlation.

**Figure 4:**
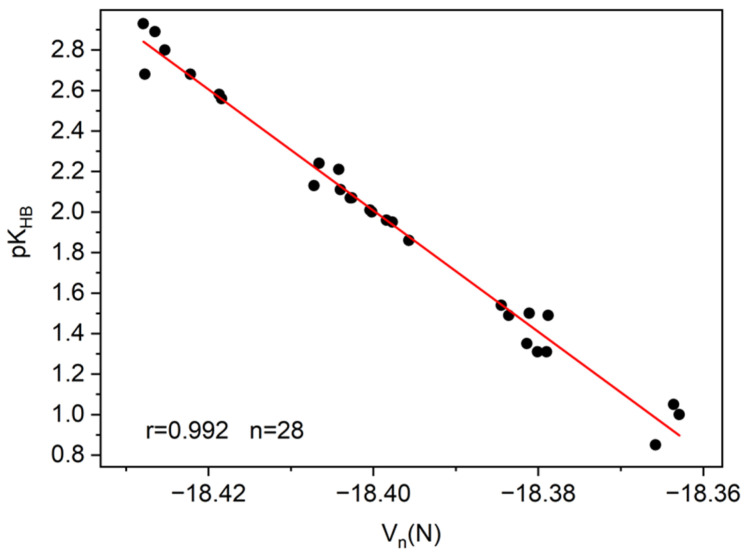
Linear correlation between the experimental hydrogen-bond basicity parameter pK_HB_ and the calculated electrostatic potential at the nucleus, *V*_n_(N), for the pyridine nitrogen atom in the pyridine series.

In these systems, the pyridine nitrogen is spatially removed from the substituent positions (3 and 4), such that *V*_n_ is predominantly governed by local electron density. Accordingly, a strong correlation between pK_HB_ and *V*_n_(N) is expected. Still, the observed relationship (Figure 4), with *r* = 0.992, is particularly remarkable.

## 3. Limits of Application

The applications across the four series of molecules considered demonstrated a clear superiority of the electrostatic potential at nuclei over partial atomic charges in predicting and explaining the variations in basicity within the different systems. Nonetheless, the definition and values of *V*_n_ are clearly system-dependent (Equation (1)), and results for molecules belonging to different classes cannot be summarized in a general correlation.

In contrast, the application of partial atomic charges to characterize basicity or other molecular properties is not subject to a priori limitations arising from the definitions of these quantities. The results, however, revealed clear system-dependent trends in the variations of the theoretical partial charges. Table 5 compares Hirshfeld charges with basicity values for selected molecules belonging to the four different classes investigated. The hydrogen-bond basicity of benzophenone (1.07) is similar to the pK_HB_ values of diethyl ether (1.01), while the oxygen Hirshfeld charges are quite different, −0.262 *e* and −0.169 *e*, respectively. The ketone and ether molecules fall within two distinctly differing ranges of partial charges. Similarly, the nitrile derivatives have nitrogen charges in the range of −0.230 *e* to −0.259 *e*, whereas in the pyridine derivatives, nitrogen charges vary from −0.153 *e* to −0.193 *e* (for the compounds included in Table 5). The pyridine nitrogen appears more basic (pK_HB_ = 1.86) than the respective value for acetonitrile (pK_HB_ = 0.91). In contrast, the partial charge at the pyridine nitrogen is −0.175 *e,* while the charge at the acetonitrile nitrogen is calculated to bear more negative atomic charges of −0.260 *e.* Within each class of compounds, however, the shifts in charges (Table 5) follow quite logical trends.

In summary, the results obtained (Table 1, Table 2, Table 3, Table 4 and Table 5) show that both the electrostatic potential at nuclei and partial atomic charges, applied as quantities characterizing basicity, perform well across the separate classes of molecules investigated. The *V*_n_ values accurately reflect the overall electrostatic environments of the respective nucleus, which is one of the key factors contributing to hydrogen bond strength. As noted above, the electrostatic potential at the nuclei is a rigorously defined quantum-mechanical quantity (Equation (1)). In contrast, atomic charge definitions necessarily involve a number of approximations [25,26,27], leading to different values for the same atomic site depending on the theoretical scheme employed. Furthermore, the *V*_n_ values inherently account for long-range intramolecular effects. The superior performance of *V*_n_, compared with partial atomic charges, in describing hydrogen-bond basicity can be attributed primarily to these factors.

In the present study, we consider three types of partial atomic charges: Hirshfeld, CM5, and NPA. Previous studies [30,39], which also examined electrostatic potential-derived charges (CHELPG and MK) as well as QTAIM charges, demonstrated that these latter types of charges exhibit only qualitative correlations with experimental molecular properties, including hydrogen-bond basicity and the σ constants of aromatic substituents.

## 4. Conclusions

Density functional theory computations at the PBE0-D3/def2-TZVPP level for representative series of ketones, ethers, nitriles, and substituted pyridines demonstrate the high effectiveness of electrostatic potential at the nucleus (*V*_n_), alongside several partial atomic charge models, in characterizing hydrogen-bond basicity at molecular reaction centers. Strong correlations between computed descriptors and experimental pK_HB_ values are observed across all classes. The systems investigated encompass both local structural modifications in the immediate vicinity of the basic site (ketones, ethers) and more remote substituent effects (nitriles, pyridine derivatives). Deviations from the general trends are identified in specific cases, most notably within the ether series.

In systems where steric hindrance limits access to the basic center—such as interactions with the proton donor (4-fluorophenol), some deviations from the correlations are observed. Similarly, in crown ethers, steric constraints and the presence of strongly polar bonds within the interaction region can lead to *V*_n_ values that somewhat depart from the overall trends.

Overall, the present results highlight the robustness of *V*_n_ as a quantitatively reliable descriptor of hydrogen-bond basicity across the four classes of molecules investigated. In all systems examined, *V*_n_ consistently exhibits stronger correlations with experimental pK_HB_ values than partial atomic charge models. At the same time, both descriptors exhibit inherent system dependence, and meaningful correlations with basicity are observed primarily within individual classes of compounds rather than across chemically diverse datasets.

## Figures and Tables

**Table 1:** Experimental hydrogen-bond basicity parameters (pK_HB_), theoretical atomic partial charges, and electrostatic potential at the nucleus for the carbonyl oxygen atom in the ketone series, obtained at the PBE0-D3/def2-TZVPP/CPCM(CCl_4_) level.

Compound	pK_HB_ ^a^	Hirsh	CM5	NPA	*V*_n_(O)
Acetone	1.18	−0.2803	−0.3315	−0.5500	−22.3786
Butan-2-one	1.22	−0.2699	−0.3223	−0.5550	−22.3786
Pentan-2-one	1.17	−0.2702	−0.3227	−0.5557	−22.3791
Cyclopentanone	1.27	−0.2859	−0.3348	−0.5463	−22.3796
Cyclohexanone	1.39	−0.2884	−0.3391	−0.5568	−22.3844
Cycloheptanone	1.41	−0.2851	−0.3376	−0.5614	−22.3852
Cyclobutanone	1	−0.2743	−0.3203	−0.5249	−22.3695
1,1,1-Trifluoropropan-2-one	−0.06	−0.2262	−0.2735	−0.4778	−22.3304
1,3-Dichloropropan-2-one	0.32	−0.2416	−0.2920	−0.5032	−22.3421
4-methoxyacetophenone	1.33	−0.2796	−0.3306	−0.5620	−22.3887
4-(Dimethylamino)acetophenone	1.76	−0.2918	−0.3424	−0.5755	−22.4035
4-tr-butylacetophenone	1.25	−0.2725	−0.3236	−0.5531	−22.3842
Acetophenone	1.11	−0.2678	−0.3191	−0.5483	−22.3789
4-Fluoroacetophenone	1	−0.2687	−0.3201	−0.5500	−22.3751
4-Chloroacetophenone	0.93	−0.2656	−0.3169	−0.5459	−22.3721
4-Cyanoacetophenone	0.97	−0.2546	−0.3063	−0.5330	−22.3568
3-Methoxyacetophenone	1.16	−0.2666	−0.3179	−0.5469	−22.3821
3-Nitroacetophenone	0.69	−0.2535	−0.3050	−0.5319	−22.3589
Benzophenone	1.07	−0.2624	−0.3112	−0.5428	−22.3794
4-(trifluormethyl)acetophenone	0.82	−0.2583	−0.3098	−0.5373	−22.3639
4-methylthioacetophenone	1.21	−0.2725	−0.3236	−0.5534	−22.3838
4-isopropylacetophenone	1.21	−0.2724	−0.3236	−0.5532	−22.3840
4-methylacetophenone	1.24	−0.2725	−0.3236	−0.5534	−22.3838
*r* (abs. value)		0.945	0.956	0.955	0.968
*R* ^2^		0.894	0.914	0.912	0.937

^a^ From ref. [31].

**Table 2:** Experimental hydrogen-bond basicity parameters (pK_HB_) and calculated partial charges (in *e*) and electrostatic potential at the nucleus (in a. u.) for the oxygen atom in the ether series, obtained with the PBE0-D3/def2-TZVPP/CPCM(CCl_4_) method.

Compound	pK_HB_^a^	Hirsh	CM5	NPA	V_n_(O)
2,2,5,5-tetramethylthetrahydrofuran	1.43	−0.1760	−0.2566	−0.5356	−22.3704
Tret-butylmethyl ether	1.19	−0.1731	−0.2724	−0.5186	−22.3590
(Diisopropyl ether)	1.11	−0.1680	−0.2637	−0.5223	−22.3620
2-methyltetrahydrofuran	1.34	−0.1820	−0.2755	−0.4990	−22.3593
Thetrahydrofuran	1.28	−0.1869	−0.2842	−0.4922	−22.3566
Tetrahydropyran	1.23	−0.1820	−0.2828	−0.5007	−22.3566
Trimethylene oxide	1.36	−0.2091	−0.2966	−0.5020	−22.3628
Dibutyl ether	0.88	−0.1680	−0.2726	−0.4992	−22.3548
Diethyl ether	1.01	−0.1695	−0.2737	−0.5040	−22.3543
(15-crown-5) ^b^	1.82	−0.1729	−0.2726	−0.5063	−22.3609
(12-crown-4) ^b^	1.73	−0.1717	−0.2717	−0.5058	−22.3567
Cyclohexene oxide	1.13	−0.2010	−0.2745	−0.4830	−22.3579
Propylene oxide	0.97	−0.2022	−0.2796	−0.4703	−22.3513
Dibenzyl ether	0.72	−0.1701	−0.2651	−0.4882	−22.3493
1,4-dioxane	1.03	−0.1790	−0.2780	−0.4924	−22.3439
2-chloroethyl ethyl ether	0.55	−0.1650	−0.2682	−0.4974	−22.3419
Dimethoxymethane	0.88	−0.1708	−0.2728	−0.4868	−22.3441
1,3-dioxane	0.93	−0.1794	−0.2770	−0.5001	−22.3445
Epichlorhydrin	0.44	−0.1877	−0.2650	−0.4543	−22.3292
1,3-dioxolane	0.75	−0.1869	−0.2792	−0.4993	−22.3431
Bis(2-chloroethyl)ether	0.27	−0.1589	−0.2625	−0.4957	−22.3175
Cyneole	1.38	−0.1713	−0.2519	−0.5513	−22.3695
2,3-dihydrofuran	0.53	−0.1581	−0.2438	−0.4688	−22.3316
(Di-tret-buthyl ether)	0.75	−0.1653	−0.2514	−0.5511	−22.3672
Ethyl vinyl ether	0.10	−0.1348	−0.2281	−0.4789	−22.3235
1,3,5-trioxane	0.50	−0.1758	−0.2699	−0.4994	−22.3287
Ethyl ethynyl ether		−0.0986	−0.1798	−0.4623	−22.2840
Dichloromethyl methyl ether	−0.09	−0.1313	−0.2324	−0.4567	−22.2893
1,1,1,3,3,3-hexafluoroisopropyl methyl ether	−0.41	−0.1566	−0.2517	−0.4708	−22.2992
Furan	−0.40	−0.0880	−0.1681	−0.3789	−22.2817
r (abs. values) (pK_HB_ vs. q_O_)		0.751	0.709	0.728	0.961
*R* ^2^		0.565	0.503	0.529	0.923

^a^ From ref. [32]. ^b^ Compounds in brackets are excluded from the correlations (see text).

**Table 3:** Experimental hydrogen-bond basicity parameters (pK_HB_) and theoretical partial charges (in *e*) and electrostatic potential at the nucleus (in a. u.) for the C≡N group nitrogen atom in the nitrile series, obtained at the PBE0-D3/def2-TZVPP/CPCM(CCl_4_) level.

Compound	pK_HB_ ^a^	Hirsh	CM5	NPA	V_n_(N)
Trichloroacetonitrile	−0.28	−0.1800	−0.3053	−0.2485	−18.3693
Dibromoacetonitrile	0.19	−0.2003	−0.3261	−0.2742	−18.3804
Cyanogen bromide	0.19	−0.2176	−0.3422	−0.3291	−18.3872
Chloroacetonitrile	0.39	−0.2207	−0.3465	−0.3119	−18.3920
p-Trifluoromethylbenzonitrile	0.54	−0.2302	−0.3545	−0.3191	−18.3976
m-Trifluoromethylbenzonitrile	0.53	−0.2313	−0.3557	−0.3207	−18.3993
o-Fluorobenzonitrile	0.64	−0.2322	−0.3562	−0.3201	−18.4078
α-Bromo-o-tolunitrile	0.69	−0.2328	−0.3586	−0.3262	−18.4066
o-Bromobenzonitrile	0.71	−0.2317	−0.3564	−0.3167	−18.4079
o-Chlorobenzonitrile	0.67	−0.2320	−0.3565	−0.3182	−18.4082
Acrylonitrile	0.7	−0.2388	−0.3629	−0.3323	−18.4074
Methyl thiocyanate	0.73	−0.2409	−0.3631	−0.3446	−18.4069
p-Fluorobenzonitrile	0.72	−0.2429	−0.3671	−0.3364	−18.4085
Phenyl cyanate	0.77	−0.2690	−0.3927	−0.4290	−18.4085
Benzonitrile	0.8	−0.2435	−0.3676	−0.3375	−18.4124
Benzyl cyanide (-−24 if)	0.81	−0.2545	−0.3802	−0.3583	−18.4172
o-Tolunitrile	0.83	−0.2447	−0.3699	−0.3428	−18.4142
Acetonitrile	0.91	−0.2590	−0.3850	−0.3658	−18.4159
Propionitrile	0.96	−0.2603	−0.3863	−0.3679	−18.4183
Butyronitrile	0.89	−0.2611	−0.3871	−0.3680	−18.4194
Trimethylsilyl cyanide	0.93	−0.2395	−0.3609	−0.3596	−18.4206
Isobutyronitrile	1	−0.2608	−0.3870	−0.3694	−18.4200
o-Methoxybenzonitrile	1.06	−0.2491	−0.3730	−0.3424	−18.4240
Hexanenitrile	0.89	−0.2619	−0.3880	−0.3691	−18.4203
Trimethylacetonitrile	0.89	−0.2614	−0.3878	−0.3693	−18.4217
p-Methoxybenzonitrile	0.97	−0.2556	−0.3795	−0.3532	−18.4219
Cyclopropyl cyanide	1.03	−0.2612	−0.3862	−0.3658	−18.4200
1-Adamantanecarbonitrile	1	−0.2642	−0.3905	−0.3691	−18.4258
p-(Dimethylamino)benzonitrile	1.23	−0.2698	−0.3933	−0.3537	−18.4355
Dimethylcyanamide	1.56	−0.3073	−0.4269	−0.4327	−18.4349
Diethylcyanamide	1.63	−0.3011	−0.4233	−0.4337	−18.4367
trans-3-Dimethylaminoacrilonitrile	1.7	−0.2993	−0.4222	−0.4089	−18.4505
N1,N1-Dimethyl-N2-cyanoformamidine	2.08	−0.3319	−0.4495	−0.4682	−18.4594
N1,N1-Dimethyl-N2-cyanoacetamidine	2.24	−0.3356	−0.4547	−0.4791	−18.4632
r (abs. value)		0.972	0.967	0.910	0.981
*R* ^2^		0.944	0.934	0.828	0.963

^a^ From ref. [33].

**Table 4:** Experimental hydrogen-bond basicity parameters (pK_HB_) and calculated atomic charges (in *e*) and electrostatic potential at the nucleus (in a. u.) for the pyridine nitrogen atom in the substituted pyridines, obtained at the PBE0-D3/def2-TZVPP/CPCM(CCl_4_) level.

Compound	pK_HB_ ^a^	Hirsh	CM5	NPA	*V*_n_(N) ^a^
4-Pyrrolidinopyridine	2.93	−0.2119	−0.4052	−0.4780	−18.4279
4-N,N-Diethylaminopyridine	2.89	−0.2111	−0.4048	−0.4752	−18.4265
4-N,N-Dimethylaminopyridine	2.80	−0.2097	−0.4033	−0.4747	−18.4253
4-Piperidinopyridine	2.68	−0.2117	−0.4052	−0.4762	−18.4277
4-(4-Methylpiperidino)pyridine	2.68	−0.2054	−0.3994	−0.4672	−18.4222
N-Methyl-N-pyridin-4-ylhydrazine	2.58	−0.2062	−0.4000	−0.4709	−18.4187
4-Aminopyridine	2.56	−0.2043	−0.3981	−0.4690	−18.4184
3,4-Dimethylpyridine	2.24	−0.1839	−0.3792	−0.4288	−18.4066
3,5-Dimethylpyridine	2.21	−0.1784	−0.3742	−0.4149	−18.4042
4-Methoxypyridine	2.13	−0.1930	−0.3876	−0.4525	−18.4072
4-tert-Butylpyridine	2.11	−0.1825	−0.3777	−0.4311	−18.4040
4-Methylpyridine	2.07	−0.1822	−0.3772	−0.4339	−18.4026
4-Ethylpyridine	2.07	−0.1820	−0.3769	−0.4329	−18.4028
3-Ethylpyridine	2.01	−0.1768	−0.3723	−0.4206	−18.4004
3-Methylpyridine	2.00	−0.1765	−0.3720	−0.4200	−18.4002
4-Phenylpyridine	1.96	−0.1777	−0.3727	−0.4266	−18.3984
4-Vinylpyridine	1.95	−0.1771	−0.3718	−0.4256	−18.3977
Pyridine	1.86	−0.1746	−0.3697	−0.4246	−18.3957
4-Chloropyridine	1.54	−0.1716	−0.3669	−0.4239	−18.3845
4-Acetylpyridine	1.50	−0.1618	−0.3571	−0.4038	−18.3811
3-Benzoylpyridine	1.49	−0.1630	−0.3586	−0.4161	−18.3788
Methyl nicotinate	1.49	−0.1673	−0.3627	−0.4229	−18.3836
3-Fluoropyridine	1.35	−0.1628	−0.3581	−0.4026	−18.3814
3-Chloropyridine	1.31	−0.1615	−0.3572	−0.4040	−18.3801
3-Bromopyridine	1.31	−0.1604	−0.3562	−0.4026	−18.3790
4-Cyanopyridine	1.05	−0.1532	−0.3486	−0.3972	−18.3636
3-Cyanopyridine	1.00	−0.1552	−0.3513	−0.4088	−18.3629
3,5-Dichloropyridine	0.85	−0.1492	−0.3456	−0.3835	−18.3658
*r* (abs. value)		0.975	0.976	0.927	0.992
*R* ^2^		0.950	0.953	0.860	0.985

^a^ From ref. [34].

**Table 5:** Hydrogen-bond basicity values (pK_HB_) ^a^ vs. Hirshfeld partial charges (in *electron* units) for selected compounds from ketone, ether, nitrile, and pyridine derivative series.

Compound	pK_HB_ ^a^	Hirsh
Acetone	1.18	−0.2803
Acetophenone	1.11	−0.2678
3-Nitroacetophenone	0.69	−0.2535
Benzophenone	1.07	−0.2624
Diethyl ether	1.01	−0.1695
1,4-dioxane	1.03	−0.1790
Ethyl vinyl ether	0.10	−0.1348
Furan	−0.40	−0.0880
Acetonitrile	0.91	−0.2604
Acrylonitrile	0.7	−0.2401
Benzonitrile	0.8	−0.2448
p-Trifluoromethylbenzonitrile	0.54	−0.2311
Pyridine	1.86	−0.1746
4-Methoxypyridine	2.13	−0.1930
4-Chloropyridine	1.54	−0.1716
4-Cyanopyridine	1.05	−0.1532

^a^ From refs. [31,32,33,34,35].

## Data Availability

The data that support the findings of this study are available in the article and its Supplementary Materials.

## Supplementary Materials

The following supporting information can be downloaded at https://www.mdpi.com/article/10.3390/molecules31142438/s1. Optimized Cartesian coordinates, electronic energies, and the number of imaginary frequencies (all reported in atomic units) for all studied structures. All computations are performed at the CPCM//PBE0-D3/Def2-TZVPP level of theory.
