# Supplementary material for: Electrostatic Potential at Nuclei vs. Atomic Charges as Descriptors of Hydrogen-Bond Basicity of Molecules
Source: Molecules. 2026 Jul 11;31(14):2438. doi: 10.3390/molecules31142438 (PMC13415106; doi:10.3390/molecules31142438)
Supplement: Supplementary file 1 [file molecules-31-02438-s001.zip › molecules-4421783-supplementary.pdf]

Electronic Supporting Information

Electrostatic Potential at Nuclei vs Atomic Charges as Descriptors of Hydrogen-Bond Basicity of Molecules

Ivan V. Atanasov,[1] Diana Cheshmedzhieva,[1]* Sonia Ilieva,[1] Boris Galabov,[1]*

and Henry F. Schaefer III[2]*

[1]Faculty of Chemistry and Pharmacy, Sofia University "St. Kliment Ohridski"
Sofia 1164, Bulgaria
[2]Center for Computational Quantum Chemistry, University of Georgia,
Athens, GA 30602, United States
E-mail: ohtdv@chem.uni-sofia.bg, galabov@chem.uni-sofia.bg, ccq@uga.edu

Ketones

Optimized Cartesian coordinates, Electronic energies and the number of imaginary frequencies (all in atomic units). The computations are performed at: CPCM//PBE0-D3/Def2TZVPP.

Acetone

0 1
C,0,-1.4450269965,-0.0022073474,-0.4386271259
C,0,0.058883156,0.0029160264,-0.4373639332
O,0,0.6973412318,0.0089498055,-1.4641030044
C,0,0.7251891041,-0.0000093108,0.910894785
H,0,-1.8148280727,-0.8858718444,0.0877386417
H,0,-1.8231755226,0.0057328047,-1.4584499521
H,0,-1.821928913,0.8680238093,0.1046852459
H,0,1.807115056,-0.0002460316,0.7989400841
H,0,0.4100913095,-0.8775722577,1.4811308247
H,0,0.4111536394,0.8764173461,1.4835274293
E = 193.010953
Nimag = 0

Butan-2-one

0 1
C,0,-1.8061150914,-0.0006914342,-0.7618489632
C,0,-0.292827133,-0.0001104189,-0.7262317968
C,0,0.2902127818,-0.0021565495,0.6661410886
C,0,1.7919729623,-0.0009305251,0.7571352307
O,0,-0.4077472525,-0.0042527154,1.6534957955
H,0,-2.1680271546,0.0005531216,-1.7907844458
H,0,-2.208075803,0.8770590159,-0.2545946418
H,0,-2.2073178451,-0.8801342579,-0.2569449945
H,0,0.1202583863,0.86909006,-1.2510686914
H,0,0.1209084097,-0.8674750199,-1.2536338458
H,0,2.1077031481,-0.0012388572,1.7980686605
H,0,2.1971838013,0.8767371619,0.2474090521
H,0,2.1983317899,-0.8777015812,0.2467345518
E = -232.291860
Nimag = 0

Pentan-2-one

0 1
C,0,-2.7330749082,-0.2950769677,0.0039155654
C,0,-1.3999775585,0.4310064064,-0.0554133045
C,0,-0.2234908164,-0.5209380096,0.0213297572
C,0,1.1291642119,0.145164766,-0.024486668
O,0,1.2528978203,1.3477319361,-0.0569863724
C,0,2.3211684806,-0.7728402283,-0.0331232387
H,0,-3.5688863226,0.4045372084,-0.0534658358
H,0,-2.8352596379,-0.859075026,0.9348449784
H,0,-2.8354768084,-1.0034117852,-0.8224804172
H,0,-1.3268222339,1.1548550154,0.7598425605
H,0,-1.3282095648,1.0136505824,-0.9774447005
H,0,-0.2601744615,-1.2601839917,-0.7883807502
H,0,-0.2613335925,-1.1157522677,0.9429880621
H,0,3.2419933089,-0.1976863145,0.0357849194
H,0,2.3243383193,-1.3563093946,-0.957751415
H,0,2.2618927637,-1.4870939295,0.7911328591
E = -271.572090

Cyclopentanone

0 1
C,0,-0.0390756645,-0.4008750869,-0.9504368528
C,0,-1.3191401554,0.028036212,-0.2231923085
C,0,-0.94612188,-0.0676063404,1.251991926
C,0,0.5370318793,0.2457091408,1.2983085287
O,0,1.1783125129,0.4727982979,2.2922591406
C,0,1.0755614408,0.2245172985,-0.1192353633
H,0,-0.0222218635,-0.0915832771,-1.9954388772
H,0,0.0487716266,-1.4909505813,-0.9289723233
H,0,-2.1818997673,-0.5824332673,-0.4895557138
H,0,-1.5582449765,1.0642312678,-0.4792371424
H,0,-1.0596998896,-1.0942023042,1.6195800901
H,0,-1.5048276464,0.5798407952,1.9277057545
H,0,1.2391796405,1.2698996356,-0.4062736845
H,0,2.0423087429,-0.2767577906,-0.1640911741
E = -270.369572
Nimag = 0

Nimag = 0

Cyclohexanone

0 1
C,0,0.8157249933,-0.6486639781,-0.8395702806
C,0,-0.6836153446,-0.9746956952,-0.7652392552
C,0,-1.452143095,0.0957603193,-0.0029510442
C,0,-0.9767744973,0.1184441177,1.4448319119
C,0,0.5298349527,0.0611411153,1.573774284
O,0,1.0714172075,-0.1297615689,2.6384067379
C,0,1.2861657536,0.2791483495,0.2938365345
H,0,1.0488223258,-0.1689621218,-1.7924204923
H,0,1.3953422944,-1.5731373463,-0.8101491528
H,0,-0.8281367091,-1.9287296196,-0.247345146
H,0,-1.0882592357,-1.1061883153,-1.770602584
H,0,-2.5264757821,-0.0958155175,-0.0393403513
H,0,-1.2965766845,1.0731214158,-0.4710152388
H,0,-1.3768611891,-0.7331160437,2.0025540796
H,0,-1.3123069373,1.0153765068,1.975118217
H,0,1.0886918216,1.3192776932,0.0073598639
H,0,2.3550801259,0.1886526888,0.4829239166
E = -309.648634
Nimag = 0

Cyclobutanone

0 1
C,0,0.3837907942,-1.0996417859,-0.091656129
C,0,-0.6686414354,-0.0000024042,-0.0775955071
C,0,0.3837885934,1.0996403838,-0.0916910151
C,0,1.4558929616,0.0000032817,0.1060387604
O,0,-1.8632032973,-0.0000017001,-0.0155633896
H,0,0.4290178101,-1.5978053733,-1.0631876978
H,0,0.2768306003,-1.8590244697,0.6841167073
H,0,0.2768211252,1.8590246832,0.6840801278
H,0,0.429016499,1.5978011823,-1.0632228051
H,0,2.2654713066,-0.0000094477,-0.6207656043
H,0,1.8802250423,0.0000216401,1.1087745524
E = -231.056904
Nimag = 0

1,3-Dichloropropan-2-one

0 1
C,0,-1.6583614775,0.229396637,-0.4417252861
Cl,0,-2.411601243,-0.4680237679,1.0092606735
C,0,-0.1578676085,0.4578681751,-0.3805076587
O,0,0.3760838997,0.9272356591,-1.3469740896
C,0,0.5609072952,0.0803537486,0.8890923241
Cl,0,2.2904937801,0.3988636575,0.8203895289
H,0,-2.1339298101,1.1889171588,-0.6404593208
H,0,-1.860403577,-0.4363563328,-1.279746707
H,0,0.4003265114,-0.9798953851,1.0916147083
H,0,0.1285062296,0.6326894498,1.7250798274
E = -1111.932150
Nimag = 0

Cycloheptanone

0 1
C,0,0.9826052519,-1.2802909633,0.1702365873
C,0,0.3117704778,-0.8392535142,-1.1296067367
C,0,-1.1277600978,-0.3658129644,-0.9669448842
C,0,-1.3237839683,0.900291871,-0.1460977899
C,0,-0.8235446409,0.8178803831,1.3037603313
C,0,0.6691223315,0.9997589363,1.3793266502
O,0,1.1476972543,2.055738649,1.7299258644
C,0,1.5755647452,-0.1483904249,0.9966378637
H,0,1.7963897194,-1.9718313832,-0.0621903254
H,0,0.2673590739,-1.8543480052,0.7686792621
H,0,0.3132792678,-1.6801013018,-1.828614776
H,0,0.9117539498,-0.0517197717,-1.6013473887
H,0,-1.7092851241,-1.1748977052,-0.5088425614
H,0,-1.561571119,-0.2027584413,-1.958025638
H,0,-2.390787548,1.1347709469,-0.1181964656
H,0,-0.8396230095,1.7482133327,-0.642279655
H,0,-1.1129009054,-0.1452891401,1.7359701738
H,0,-1.2709597357,1.6144454284,1.8986217729
H,0,2.4351789188,0.2944269149,0.4866041577
H,0,1.9641121583,-0.539851847,1.9443865579
E = -348.930511
Nimag = 0

1,1,1-Trifluoropropan-2-one

0 1
C,0,-1.1893941011,-0.3621551547,0.4656787576
F,0,-1.5309512056,-0.7373535444,-0.7737565145
F,0,-2.1889958628,0.3314842367,0.9818172659
F,0,-1.0247310742,-1.4731228801,1.1954572986
C,0,0.1317473015,0.4538307473,0.4312438625
O,0,0.133574819,1.5749097182,0.8507203274
C,0,1.3032742578,-0.2687297929,-0.1407404195
H,0,2.1767537413,0.3776546472,-0.1240054329
H,0,1.0818503319,-0.5804869739,-1.1639945373
H,0,1.4934537923,-1.1778670035,0.4342523923
E = -490.596538
Nimag = 0

4-methoxyacetophenone

0 1
C,0,-2.2551269007,1.3586686071,0.0185937037
C,0,-1.0490905071,0.5090978428,-0.1317106202
C,0,-1.1165305015,-0.8751464759,-0.3167624123
C,0,0.0291511581,-1.6287129604,-0.4523662864
C,0,1.2816482121,-1.0128998414,-0.40608745
C,0,1.3670368319,0.3682701687,-0.2219393893
C,0,0.2073294081,1.1086128597,-0.0876967333
O,0,-2.159319813,2.559973503,0.1782546161
C,0,-3.6077186502,0.6992884383,-0.0309105618
O,0,2.3431690598,-1.8259533806,-0.5472304831
C,0,3.6354254074,-1.2551568452,-0.508679413
H,0,-2.0753925499,-1.3763078582,-0.3562185434
H,0,-0.0170605553,-2.7008455607,-0.5959004461
H,0,2.3257309541,0.8661006536,-0.1828800347
H,0,0.2603536086,2.1807793566,0.0556402986
H,0,-4.3772746672,1.457149783,0.0939895321
H,0,-3.7016552967,-0.0497993035,0.7585544408
H,0,-3.7526598591,0.1855537091,-0.9839448857
H,0,4.3327687293,-2.0789148129,-0.6410154626
H,0,3.82732349,-0.7699497418,0.452371313
H,0,3.7744524411,-0.5307021414,-1.3159671824

4-(Dimethylamino)acetophenone

0 1
C,0,-2.2824111561,1.5104766165,1.3481914673
C,0,-1.0785525649,0.752838587,0.9610571673
C,0,-1.1298426494,-0.4849042999,0.3168941892
C,0,0.0157187604,-1.1680204823,-0.0321180498
C,0,1.2897588508,-0.6349813991,0.2531306728
C,0,1.3396998706,0.6180613363,0.903815634
C,0,0.18454952,1.281702893,1.2426024713
O,0,-2.1997872803,2.5891882886,1.9089869735
C,0,-3.6346232891,0.9212570183,1.0347330594
N,0,2.4267357897,-1.3005343051,-0.0846610786
C,0,3.717396953,-0.7267698697,0.2062583841
C,0,2.3482185105,-2.5726013265,-0.7584290383
H,0,-2.0856746032,-0.9345920642,0.0771428956
H,0,-0.0773644264,-2.122536812,-0.5301393796
H,0,2.2920819496,1.0696761783,1.1428731361
H,0,0.2381796617,2.2417748913,1.7414832148
H,0,-4.4073564682,1.6014137241,1.3852230543
H,0,-3.7570529257,-0.0496260498,1.5200815242
H,0,-3.7492852481,0.7633644712,-0.0400446563
H,0,4.495280458,-1.4047301925,-0.1360241033
H,0,3.8577464728,-0.5678105869,1.2804430061
H,0,3.8595497431,0.2338986712,-0.300108202
H,0,3.3535422767,-2.9453198388,-0.9378280403
H,0,1.8404696756,-2.490605274,-1.7255728287
H,0,1.8150641189,-3.317060175,-0.1578794729
E = -518.464461
Nimag = 0

Acetophenone

0 1
C,0,-1.2613211021,1.3515555335,0.061357152
C,0,-0.049720721,0.5621818824,-0.3065692784
C,0,-0.0771265182,-0.8250336692,-0.439537239
C,0,1.0732706566,-1.5190214542,-0.783142694
C,0,2.2597491786,-0.8322249417,-0.9965964688
C,0,2.2957622563,0.5514958228,-0.8661277746
C,0,1.1482733401,1.2431761556,-0.5233829909
O,0,-1.20367168,2.5584711043,0.1694883913
C,0,-2.5533842298,0.6182447861,0.2945973475
H,0,-0.9961939529,-1.3731156171,-0.2758595697
H,0,1.0433540503,-2.5970835817,-0.8843489158
H,0,3.1584464929,-1.3747899508,-1.2650910953
H,0,3.2221388432,1.0877839503,-1.0328148096
H,0,1.1553395551,2.3206505461,-0.4167188559
H,0,-3.3285778137,1.3358322523,0.5518514155
H,0,-2.444031939,-0.1079285618,1.1032584232
H,0,-2.8491754162,0.064900743,-0.5995970374
E = -384.594352
Nimag = 0

4-Chloroacetophenone

0 1
C,0,-1.4994386578,1.3571765485,-0.0650407299
C,0,-0.2803161584,0.5623469548,-0.3899922708
C,0,-0.2994125232,-0.826641711,-0.4964320842
C,0,0.8539822051,-1.5306413165,-0.7999042688

E = 499.045558
Nimag = 0

4-tr-butylacetophenone

0 1
C,0,-2.3647695739,1.9042975599,1.157681743
C,0,-1.1547038951,1.0980367037,0.8419551114
C,0,-1.1894781792,-0.2845418531,0.6974883938
C,0,-0.0356355137,-0.9970129087,0.4027332923
C,0,1.1892299446,-0.355525752,0.2424278568
C,0,1.2123831139,1.0363252509,0.3899682819
C,0,0.0693209372,1.7494855367,0.6828477449
O,0,-2.2939006609,3.1104941606,1.2769288277
C,0,-3.6791196951,1.1914569643,1.32589001
C,0,2.4765500958,-1.1030346724,-0.0812595357
C,0,3.4982878822,-0.852362786,1.0345261369
C,0,3.0382465198,-0.5855364364,-1.4110980889
C,0,2.2587786791,-2.6083422209,-0.2045140212
H,0,-2.1206084839,-0.8250349935,0.8135055589
H,0,-0.105850876,-2.0711345834,0.2986887219
H,0,2.1458628017,1.5736824135,0.2729707412
H,0,0.1009269155,2.8261693964,0.7944539728
H,0,-4.4534396857,1.9206323469,1.5514971228
H,0,-3.6198609593,0.4589826157,2.1340306649
H,0,-3.9422160583,0.648285244,0.4154622052
H,0,4.4302236318,-1.3777382989,0.8121185368
H,0,3.1229632027,-1.2143409306,1.9941287802
H,0,3.7296643089,0.2084308875,1.141352609
H,0,3.9665396928,-1.108738601,-1.6530058478
H,0,3.2571316527,0.4824817789,-1.3703609564
H,0,2.3293927135,-0.7540919386,-2.2246386027
H,0,3.2084869788,-3.0945839044,-0.4361782967
H,0,1.5580017321,-2.8521909923,-1.0059578643
H,0,1.883640778,-3.0410599866,0.7254809009
E = -541.721003
Nimag = 0

4-Fluoroacetophenone

0 1
C,0,-1.3177538318,1.3517694502,0.0810820725
C,0,-0.108205679,0.5634018067,-0.2847246181
C,0,-0.1323423325,-0.8241871447,-0.4171676779
C,0,1.0128221414,-1.5244231565,-0.7586133237
C,0,2.1796943617,-0.8141319418,-0.9648339712
C,0,2.2424954888,0.5632847575,-0.8427916999
C,0,1.0905197448,1.2444382735,-0.5015364197
O,0,-1.2594262813,2.5590412504,0.1884242285
C,0,-2.6099902394,0.6183580093,0.3135592761
F,0,3.2897748243,-1.4817880728,-1.2949152772
H,0,-1.0493204247,-1.3753643337,-0.2544794347
H,0,1.012185203,-2.6011318052,-0.8655886824
H,0,3.1802939851,1.0752999448,-1.0145611693
H,0,1.0984343148,2.3217910484,-0.3961383579
H,0,-3.385121028,1.3363284352,0.569753667
H,0,-2.5022578462,-0.1069071697,1.1232935115
H,0,-2.9059324009,0.0651576484,-0.5807101235
E = -483.788332
Nimag = 0

4-Cyanoacetophenone

0 1
C,0,-1.8865420462,1.361105507,0.3929040663
C,0,-0.6759125721,0.5728591551,-0.0025113141
C,0,-0.708738567,-0.8106738676,-0.1618380719
C,0,0.4313446217,-1.5028545916,-0.5281473652

C,0,2.0358391119,-0.833532508,-0.9980585538
C,0,2.0806152094,0.55142192,-0.8975603325
C,0,0.9219852152,1.238929402,-0.5942202552
O,0,-1.4421155984,2.565815543,0.0189989381
C,0,-2.7948418548,0.6267210301,0.1546267947
Cl,0,3.4787478029,-1.7022224618,-1.3768369503
H,0,-1.2180985604,-1.3779084871,-0.343181029
H,0,0.8407684619,-2.6091569785,-0.8828410722
H,0,3.0141965506,1.074663428,-1.0563206119
H,0,0.9295539613,2.3182484545,-0.5099577257
H,0,-3.576390206,1.3478936629,0.3804235064
H,0,-2.7024126698,-0.0825530017,0.980301365
H,0,-3.0706572897,0.0553125207,-0.7346467199
E = -844.063014
Nimag = 0

3-Methoxyacetophenone

0 1
C,0,-2.3209263545,1.4138785343,0.6115144612
C,0,-1.1395778202,0.5700944036,0.2579785943
C,0,-1.2289743535,-0.8172624003,0.1259611522
C,0,-0.0985839921,-1.5428316888,-0.203337393
C,0,1.1231348137,-0.912753186,-0.4045596696
C,0,1.2122647694,0.4722330297,-0.2728159625
C,0,0.07808545,1.2047899529,0.0581166266
O,0,-2.2136199816,2.6168671081,0.7197141284
C,0,-3.6449875151,0.7354643466,0.8295555636
O,0,2.3481017141,1.1818151586,-0.4463603078
C,0,3.5244798534,0.4803602062,-0.7839241164
H,0,-2.1692114916,-1.3296884083,0.2777527582
H,0,-0.1592959385,-2.6194498149,-0.3076513129
H,0,1.9909542158,-1.5043352545,-0.6611235876
H,0,0.1440932451,2.2803044815,0.1611707331
H,0,-4.3926068425,1.4849057135,1.0773698525
H,0,-3.5747853775,0.0057502193,1.6392789035
H,0,-3.9520380277,0.1939842699,-0.0680097979
H,0,4.3079328703,1.2291151803,-0.8757588442
H,0,3.4171491912,-0.0464577181,-1.7370437557
H,0,3.8013685717,-0.2359421338,-0.0043450261
E = -499.042693
Nimag = 0

Benzophenone

0 1
C,0,0.1154236182,1.0199898109,-0.0681897188
C,0,1.1886522681,0.3949675679,-0.8914744231
C,0,1.2902331361,-0.9850411702,-1.0583277523
C,0,2.3463450935,-1.5247399524,-1.776942557
C,0,3.2950475648,-0.6898280056,-2.3498616865
C,0,3.1986676856,0.6874480666,-2.1881406761
C,0,2.1584950823,1.2256331417,-1.4515624229
O,0,0.3331095918,2.0507187687,0.5392350042
C,0,-1.2259781958,0.3755325727,0.0069578089
C,0,-1.9817198697,0.5588044515,1.1643693832
C,0,-3.250291334,0.0157018677,1.2638537448
C,0,-3.7874405379,-0.6939698474,0.1960491048
C,0,-3.0504903663,-0.8618061891,-0.9675895381
C,0,-1.7697610753,-0.3380260372,-1.0596588828
H,0,0.5556701957,-1.6392517028,-0.6056048026
H,0,2.4284192612,-2.598974589,-1.8893181487
H,0,4.1138222547,-1.1121234098,-2.9202138707
H,0,3.939961924,1.3394127312,-2.6341238649
H,0,2.0780490713,2.2948344186,-1.2996822046
H,0,-1.5547367049,1.1302794586,1.9791309365

C,0,1.6200725602,-0.8056161581,-0.7383391602
C,0,1.663492253,0.5818548194,-0.5811815902
C,0,0.5188795306,1.2592950705,-0.2155360995
O,0,-1.8135032256,2.5632138107,0.5201650308
C,0,-3.1759654438,0.6276249839,0.6236821642
C,0,2.7997145389,-1.5146188714,-1.1164852647
N,0,3.7515615421,-2.0868603802,-1.4216338731
H,0,-1.6258091318,-1.3617296843,-0.0013064959
H,0,0.4073741215,-2.577474966,-0.6522639845
H,0,2.5915238461,1.1131733381,-0.7468651749
H,0,0.5286295136,2.3339649081,-0.0878789527
H,0,-3.9473508224,1.3412372298,0.9017639749
H,0,-3.0601760582,-0.1140582498,1.4173988996
H,0,-3.4807586605,0.0923039465,-0.2785747891
E = -476.766904
Nimag = 0

3-Nitroacetophenone

0 1
C,0,-1.8715389076,1.3470509307,0.0678860553
C,0,-0.6500564783,0.5480843058,-0.266832646
C,0,-0.6756655483,-0.8363515486,-0.4210123192
C,0,0.4817641045,-1.5367323621,-0.731513945
C,0,1.6796810323,-0.8635157329,-0.8922812959
C,0,1.6890394841,0.5143321311,-0.7354447047
C,0,0.5518844375,1.2300784402,-0.4269339048
O,0,-1.7959777564,2.5488750425,0.1906952637
C,0,-3.1740196797,0.621777506,0.2441557463
N,0,2.9565805762,1.2360832888,-0.9043375823
O,0,2.9435820173,2.4391556067,-0.7632878352
O,0,3.9422048853,0.5831669101,-1.1753042044
H,0,-1.6030563888,-1.3808719207,-0.2996114944
H,0,0.4499282381,-2.6120952625,-0.849317044
H,0,2.5959949508,-1.3827904083,-1.1343056959
H,0,0.5841286575,2.3040725136,-0.3097315472
H,0,-3.9531494064,1.3416316181,0.4814203067
H,0,-3.0984985254,-0.1141624448,1.0480572594
H,0,-3.4401036928,0.0813203861,-0.6672034124
E = -588.981767
Nimag = 0

4-(trifluormethyl)acetophenone

0 1
C,0,-1.8236033715,1.396083366,0.6648359376
C,0,-0.602553976,0.6038433512,0.3173448818
C,0,-0.6453462197,-0.7700175878,0.0959284591
C,0,0.5090027278,-1.4659117762,-0.224560744
C,0,1.7126270496,-0.7852454352,-0.3265300552
C,0,1.7693705894,0.5865184673,-0.1070626737
C,0,0.615172856,1.2742303115,0.2122370071
O,0,-1.7444391926,2.5912141089,0.8467533265
C,0,-3.136660569,0.6761989057,0.7815183425
C,0,2.9773967882,-1.5369496531,-0.6274530319
F,0,3.6277509568,-1.8713358208,0.5004239988
F,0,3.8288954849,-0.8111024689,-1.3631146329
F,0,2.7450572533,-2.6745161926,-1.2929296405
H,0,-1.579799526,-1.310462493,0.1693924247
H,0,0.4718016951,-2.5328670483,-0.400230889
H,0,2.7125857277,1.1105433387,-0.192912807
H,0,0.6351297602,2.3426218217,0.3840421909
H,0,-3.9134718329,1.3912718379,1.0402214644
H,0,-3.0831648093,-0.1000750508,1.5482965319
H,0,-3.3907473921,0.1843740177,-0.1602770908

H,0,-3.8259447452,0.1481148913,2.1719093107
H,0,-4.7839031708,-1.1129052489,0.2707542767
H,0,-3.473410042,-1.4015678292,-1.8061256647
H,0,-1.1999577055,-0.4636177657,-1.9718443562
E = -576.175180
Nimag = 0

4-methylthioacetophenone

0 1
C,0,-2.375813534,1.4436356202,-0.132755731
C,0,-1.1618992325,0.592382398,-0.2143997561
C,0,-1.2181137938,-0.7834444041,-0.4480636388
C,0,-0.0651020282,-1.5378973389,-0.5167332683
C,0,1.186615247,-0.9351147397,-0.3525718194
C,0,1.2504052079,0.4403603298,-0.1185562803
C,0,0.0887041862,1.1849925352,-0.052092864
O,0,-2.2868613146,2.6381854726,0.0710445675
C,0,-3.7213038285,0.793023329,-0.3084641937
S,0,2.5794523408,-1.9845832802,-0.4585043722
C,0,3.9682941778,-0.8805246832,-0.2037115464
H,0,-2.1715120399,-1.2793531653,-0.5793062498
H,0,-0.1281178977,-2.6045366064,-0.6989861477
H,0,2.2024205614,0.9357085915,0.0125230057
H,0,0.133835784,2.2518071743,0.1291517115
H,0,-4.4967406868,1.5498729271,-0.2192349191
H,0,-3.8777115094,0.0189545121,0.4462295588
H,0,-3.7922071637,0.3111368527,-1.2862430254
H,0,4.8554786068,-1.5097988953,-0.2647528399
H,0,3.9327658705,-0.4151185237,0.7810083727
H,0,4.0211720468,-0.1194701058,-0.9818835641
E = -821.947456
Nimag = 0

4-methylacetophenone

0 1
C,0,1.6924911947,-0.2026254066,-0.017994771
C,0,0.211274743,-0.0584972884,-0.0077232445
C,0,-0.4210127304,1.1818937115,0.0283436176
C,0,-1.8043586641,1.2671292074,0.0384656763
C,0,-2.5935143531,0.1211833078,0.0114754453
C,0,-1.9540903069,-1.1201015218,-0.0217042571
C,0,-0.5769771204,-1.2101561374,-0.0319459648
O,0,2.2098702159,-1.3006009446,-0.0471766098
C,0,2.5358517691,1.0432316901,0.0082557331
H,0,0.1617950798,2.094102511,0.0512430455
H,0,-2.2801978668,2.2409468782,0.0696346078
H,0,-2.5518263497,-2.0250596587,-0.0376226429
H,0,-0.0826019226,-2.1733105393,-0.0565305549
H,0,3.5861039542,0.7623826963,-0.0064031877
H,0,2.3272098277,1.631119288,0.9049512758
H,0,2.3146586064,1.6772723632,-0.8532520974
C,0,-4.087696524,0.2082310994,-0.0002206493
H,0,-4.4334666559,1.2038097716,0.2780439488
H,0,-4.4778699939,-0.013972653,-0.9973735838
H,0,-4.5302574827,-0.5160467647,0.6861370129
E = -423.879689
Nimag = 0

E = -721.475102
Nimag = 0

4-isopropylacetophenone

0 1
C,0,-2.3643692238,1.4184171373,1.1487598844
C,0,-1.1304224465,0.6480490225,0.8340364412
C,0,-1.128199081,-0.7366669341,0.6875697897
C,0,0.0472959673,-1.4104299,0.3941215585
C,0,1.2490396675,-0.7270910898,0.2387939312
C,0,1.2400284617,0.6620683592,0.3869709407
C,0,0.0730017063,1.3382284307,0.6791099739
O,0,-2.3292839768,2.625970142,1.2698897143
C,0,-3.6578292708,0.667662535,1.313687989
C,0,2.5233509473,-1.4682198257,-0.0804205428
C,0,3.5589998284,-1.2996147329,1.0289937522
C,0,3.0939060421,-1.0400531007,-1.4305766921
H,0,-2.0444394223,-1.3023436001,0.8012321654
H,0,0.0319457559,-2.4892191143,0.2832879826
H,0,2.1616883404,1.2210722388,0.2714722661
H,0,0.0683630811,2.415090967,0.7935557835
H,0,-4.4529716972,1.3742074148,1.538775735
H,0,-3.579131012,-0.063694893,2.1211625974
H,0,-3.9038690404,0.1183788859,0.402180315
H,0,2.2701368029,-2.5312181036,-0.1447193621
H,0,4.4542071082,-1.884310041,0.80646285
H,0,3.1655028029,-1.6286413006,1.9924189393
H,0,3.8601404988,-0.2540985767,1.1285719731
H,0,3.9860436293,-1.6230671552,-1.6692572533
H,0,3.3784035253,0.0147641609,-1.4192383826
H,0,2.3667050053,-1.1828239264,-2.2318763496
E = -502.441109
Nimag = 0

Ethers

Optimized Cartesian coordinates, Electronic energies and the number of imaginary frequencies (all in atomic units). The computations are performed at: CPCM//PBE0-D3/Def2TZVPP.

2,2,5,5-tetramethylthetrahydrofuran

0 1
C,0,0.6845781985,-0.0174849472,-0.0555926166
O,0,2.1172434048,0.007610487,0.0014842786
C,0,2.6319006592,1.3449776772,0.0561029218
C,0,1.4059732337,2.2221695509,0.3238114326
C,0,0.2810911097,1.434506502,-0.325797154
H,0,1.2406788994,2.3141103469,1.4000543748
H,0,1.5234110472,3.2277111869,-0.0815372807
H,0,-0.7041785028,1.6694358528,0.0782626225
H,0,0.2523982295,1.619816631,-1.4023220446
C,0,3.6598748554,1.410848947,1.1696848578
H,0,4.4873738067,0.7281880946,0.9667263535
H,0,4.0642185366,2.4213257576,1.2611002198
H,0,3.2055373359,1.1294036574,2.1210859105
C,0,3.2745788751,1.6709578906,-1.2862821554
H,0,3.7428019844,2.6575151784,-1.2645880498
H,0,4.0398430589,0.9288104144,-1.5203044596
H,0,2.5332578015,1.6577700598,-2.0873647296
C,0,0.1558537379,-0.5077884525,1.286561782
H,0,-0.9313870121,-0.609547913,1.2632348547
H,0,0.5904092464,-1.480741633,1.5225652157
H,0,0.4210532483,0.185187811,2.0871383475
C,0,0.2721437478,-0.9620256177,-1.168642945
H,0,0.6294631402,-1.9731323717,-0.9637895829
H,0,-0.8155616906,-0.9956025678,-1.2618139182
H,0,0.6938389184,-0.6329309327,-2.1197862356
E = -389.41789
Nimag = 0

Tret-buthylmethyl ether

0 1
C,0,-2.9257349964,0.2347627193,-0.0748631067
C,0,-2.4702593747,0.9018073823,-1.3678077793
H,0,-2.7654898665,0.2942348912,-2.2250191255
H,0,-2.9165644122,1.8892962432,-1.4966253808
H,0,-1.3835846441,1.0060278712,-1.376709031
C,0,-4.4472517155,0.1623867822,-0.0127862573
H,0,-4.8233348668,-0.4753527651,-0.8146386746
H,0,-4.7667832902,-0.25939605,0.9420962387
H,0,-4.9076064347,1.1446319112,-0.1318889236
C,0,-2.3327169561,-1.1601054381,0.0291927955
H,0,-1.2428422911,-1.1076947317,0.0096046474
H,0,-2.6379961407,-1.6294886807,0.9659119298
H,0,-2.6675919519,-1.7848154251,-0.8002170936
O,0,-2.3970443474,0.9149522584,1.0677266407
C,0,-2.7728698827,2.2543876924,1.2498306706
H,0,-2.277554564,2.5954300744,2.1591054025
H,0,-2.4509632539,2.8986280656,0.4239035774
H,0,-3.8537205713,2.3744155192,1.3846667297
E = -272.760209
Nimag = 0

(Diisopropyl ether)

0 1
C,0,-3.0800472445,0.5700067803,0.1557307596
H,0,-2.6017299848,1.283316749,-0.5298348313
C,0,-2.7316363422,-0.8431064543,-0.2798375935
H,0,-3.1705067595,-1.5645336592,0.4130766143
H,0,-3.110146053,-1.0558782735,-1.2812648311
H,0,-1.6493078907,-0.9879878835,-0.2926019702
C,0,-2.610761272,0.8603039864,1.5644136441
H,0,-1.5327318218,0.7133537318,1.6509014337
H,0,-2.8480258275,1.8874444122,1.8444892383
H,0,-3.1072847388,0.1884805208,2.2683952426
O,0,-4.4848714257,0.7693993565,0.1351855917
C,0,-5.030882472,1.1115900593,-1.1291432599
H,0,-4.4919702999,0.5671806024,-1.9170498577
C,0,-6.4747001336,0.6596612381,-1.126367811
H,0,-7.0196650535,1.158469174,-0.321598678
H,0,-6.9586866635,0.9054511082,-2.0732398343
H,0,-6.5398532425,-0.4176925434,-0.9687914456
C,0,-4.9026008622,2.603838254,-1.3836759176
H,0,-3.8606960836,2.9266260689,-1.3431723885

2-methyltetrahydrofura

0 1
C,0,0.0635710209,-0.4650098899,-0.031919116
O,0,1.3007642307,-0.4063532845,0.6490742624
C,0,1.9261689163,0.7915315898,0.2147114994
C,0,0.7959032918,1.8198227632,0.1980450943
C,0,-0.4561322866,0.9785524609,-0.0988076555
H,0,0.213181114,-0.868100393,-1.0432234078
H,0,-0.5916898628,-1.14402758,0.5142404868
H,0,2.2937936895,0.6425274418,-0.8140085038
H,0,0.7225779607,2.2926294995,1.1799537555
H,0,0.9691851916,2.6052047868,-0.5382598095
H,0,-1.2332917375,1.1542312517,0.645087724
H,0,-0.882955495,1.1990166247,-1.0776776563
C,0,3.081880168,1.1122297458,1.1251940562
H,0,3.8217423026,0.3105525504,1.1140984933
H,0,3.5710660733,2.0342956189,0.8058760568
H,0,2.7277814125,1.2451553038,2.1498003002
E = -271.561031
Nimag = 0

H,0,-5.2959926348,2.8632422104,-2.3688377021
H,0,-5.4632745438,3.1592960617,-0.6284888336
E = -312.047135
Nimag = 0

Thetrahydrofuran

0 1
C,0,1.1238930615,-0.4531469878,-0.1420614293
O,0,0.0000062087,-1.1715012796,0.3272094205
C,0,-1.123889701,-0.4531615753,-0.1420620168
C,0,-0.7708605569,1.0255903856,0.0341400997
C,0,0.7708444876,1.0256005063,0.0341396499
H,0,1.9954156143,-0.7717047242,0.4303036422
H,0,1.2998281222,-0.684881463,-1.2025479412
H,0,-1.2998207414,-0.6848976432,-1.2025488427
H,0,-1.9954084945,-0.771730958,0.4303022854
H,0,-1.1913664075,1.6378643179,-0.7639293298
H,0,-1.1582614889,1.4035541045,0.980493603
H,0,1.1913417931,1.6378791458,-0.7639306705
H,0,1.1582411026,1.4035701711,0.9804925297
E = -232.274057
Nimag = 0

Trimethylene oxide

0 1
C,0,2.0114725357,0.2790172679,-0.0000101037
C,0,2.0835850314,1.8087618908,-0.0001290199
C,0,0.5538451704,1.7365531437,-0.0003764153
H,0,2.4094989937,-0.2157474328,-0.8907111915
H,0,2.4094238086,-0.2156408711,0.8907807256
H,0,2.5285257585,2.2535079909,-0.8879874664
H,0,2.5281932888,2.253620955,0.8878401104
H,0,0.0592807517,2.1343243295,-0.891302594
H,0,0.0589373086,2.1346974171,0.8901893811
O,0,0.5738829526,0.2989646091,-0.0000764263
E = -192.962542
Nimag = 0

Tetrahydropyran

0 1
C,0,-0.5825315439,-0.6905735387,0.0214330981
H,0,-0.3038884323,-1.7511356713,0.0145352437
H,0,-1.6738031849,-0.6504032328,-0.0124142341
C,0,-0.0546107126,-0.0427712111,1.2949091272
H,0,-0.4349204876,0.9805899793,1.3762207898
H,0,-0.3887567675,-0.5866452977,2.1827045897
C,0,0.0241470912,-0.0056406506,-1.1962371473
H,0,-0.252323684,-0.522642438,-2.1192542022
H,0,-0.3516263771,1.0198854733,-1.2710864151
C,0,1.4622443249,0.0008696879,1.2672624969
H,0,1.8612594603,0.5375988317,2.1296392561
H,0,1.8640525404,-1.024884567,1.2920468104
C,0,1.5362197274,0.0365926732,-1.071591865
H,0,1.9403529327,-0.9880996584,-1.1021545733
H,0,1.9879396111,0.599204485,-1.8903737923
O,0,1.9494103118,0.6700517551,0.1218435075
E = -271.561749
Nimag = 0

Dibuthyl ether

0 1
C,0,-3.0176613608,-0.5800941419,0.0422580372
H,0,-2.648624464,-1.6087584402,0.0257466181
H,0,-2.6453869908,-0.082782135,-0.8570213416
H,0,-4.1068219365,-0.6157078158,-0.0232975059
C,0,-2.5564454853,0.144064406,1.295749779
H,0,-2.9641145502,1.1607918964,1.3035117415
H,0,-2.9673039747,-0.354776607,2.1802505055
C,0,-1.0419817964,0.2073085206,1.4105874616
H,0,-0.6225073219,0.7146445471,0.5358022214
H,0,-0.6257073822,-0.8052593408,1.4150543336
C,0,-0.5849165384,0.9272285029,2.6567320327
H,0,-0.9798801626,0.4244083692,3.5536056886
H,0,-0.9766418626,1.9566203185,2.6672330283
O,0,0.8206850705,0.9456144168,2.6936503944
C,0,1.3287046517,1.6014056076,3.8291110537
H,0,0.9639312702,1.1092304093,4.7444856071
H,0,0.967174725,2.6414355507,3.8581011996
C,0,2.8378782109,1.5741898793,3.7875876248
H,0,3.1742952112,2.0522497597,2.8619093119
H,0,3.1710787889,0.5323543534,3.7411760246
C,0,3.4669895998,2.2661478631,4.9860035733
H,0,3.1157636884,1.7883330573,5.9070476953
H,0,3.1189706115,3.3038907311,5.0302902774
C,0,4.985544259,2.2394591763,4.9454241466
H,0,5.3624462919,2.7383972637,4.0489774517
H,0,5.4194275838,2.7404164699,5.812963006
H,0,5.3592171296,1.2124281541,4.9317578774
E = -390.600199
Nimag = 0

Diethyl ether

0 1
C,0,-3.0927259742,0.263668321,0.0172961983
H,0,-2.7204726108,-0.7621282853,0.0117888627
H,0,-2.7176103844,0.7699916417,-0.873650614
H,0,-4.1824468263,0.2351517174,-0.0355675184
C,0,-2.6461789424,0.9835533297,1.2643880214
H,0,-3.0348139557,2.0130449306,1.276622839
H,0,-3.0376913725,0.4812720819,2.1618696924
O,0,-1.2392709272,1.001988376,1.3008519505
C,0,-0.7311626449,1.6580997069,2.4376912179
H,0,-1.0979633648,1.1645726234,3.3503406603
H,0,-1.0950616907,2.696305476,2.4650246894
C,0,0.775257013,1.6260586093,2.387194899
H,0,1.1402923349,2.1287699312,1.4900371613
H,0,1.1956218043,2.1294644418,3.2595338896
H,0,1.1373902638,0.5966969492,2.3755577318
E = -233.479085
Nimag = 0

(12-crown-4)

0 1
C,0,-1.4642133908,3.1377393952,0.1922953715
H,0,-0.802084606,2.2643613083,0.266445059
H,0,-1.3873869407,3.5359809929,-0.83136703
C,0,-0.9998836439,4.1925798096,1.1663452817
H,0,0.0030242801,4.513134274,0.868718908
H,0,-1.6562859741,5.0694265924,1.1126735864
O,0,-2.7847294972,2.7631604423,0.4922131362
O,0,-0.9200967013,3.7026153956,2.4834362185
C,0,-3.2605288496,1.6925735778,-0.2860178481
H,0,-3.660707241,2.0577701076,-1.2427499074
H,0,-2.4484244288,0.9882828727,-0.5073939603
C,0,-4.3348903794,0.9601018952,0.4742452892
H,0,-4.8721663866,0.2942394765,-0.2130518469
H,0,-5.0568868289,1.6852978535,0.8653473682
C,0,-2.0391835785,3.9838681987,3.2941381933

(15-crown-5)

0 1
C,0,-2.4851986018,3.3627938216,0.3896769995
H,0,-1.5088497813,3.403430375,-0.1154956863
H,0,-3.2211999731,3.8598189872,-0.259898675
C,0,-2.4067465287,4.0907351828,1.7038443476
H,0,-2.3873471269,5.1745985024,1.5237738463
H,0,-3.2999470934,3.848393843,2.2878005516
O,0,-2.8640807648,2.0397556302,0.6472565018
O,0,-1.2388491537,3.6917767789,2.3894946656
C,0,-2.8789665287,1.2020436955,-0.4743936214
H,0,-3.5108809093,1.6200725799,-1.2719517285
H,0,-1.8641840576,1.0758412435,-0.8806057935
C,0,-3.4177392824,-0.1368467557,-0.0502988951
H,0,-2.9256123612,-0.4286818723,0.8825384882
H,0,-3.1869965301,-0.8869636005,-0.8194852081
C,0,-1.2896124777,3.8343461248,3.7888702429
H,0,-2.0237411218,4.5944806843,4.0814337334
H,0,-0.3061577623,4.1794153111,4.1231334137
C,0,-1.5911744823,2.5333040201,4.491693308
H,0,-0.8861014029,1.7648712485,4.1440694408
H,0,-1.4346943467,2.6684254468,5.5739086076
O,0,-4.8146526653,-0.0417239011,0.1310476103
O,0,-2.9113996916,2.1351898962,4.2268506327
C,0,-5.368159928,-0.9843075923,1.0178171369
H,0,-6.3301843932,-1.3036303066,0.6048080772
H,0,-4.7308287354,-1.8730619541,1.0968390746
C,0,-5.6178701457,-0.4092327793,2.3904972529
H,0,-6.2160373441,-1.1273828636,2.9739269142
H,0,-6.2052057727,0.5147584324,2.2918129557
C,0,-3.2412358139,0.9090638191,4.8289504456
H,0,-2.4730191164,0.1533072203,4.6138179646
H,0,-3.3002391692,1.025202425,5.9212423
C,0,-4.5661775276,0.4245042104,4.3072872215
H,0,-5.2662881443,1.2704808102,4.2655563786
H,0,-4.9813727055,-0.3198555467,5.0027109534
O,0,-4.3979814807,-0.1421438469,3.0324177528
E = -768.610210
Nimag = 0

Cyclohexene oxide

0 1
C,0,-1.3034127277,-0.5525294033,-0.5117128839
C,0,-0.3669691391,-1.415386128,0.3248926304
C,0,-0.1147089001,1.5871747845,0.1024403725
C,0,-1.4415642646,0.8335328486,0.1002562291
H,0,-0.8384636567,-1.6504150954,1.2858761421
H,0,-0.1682744217,-2.3713018599,-0.1690867488
H,0,-0.9126060243,-0.4687713097,-1.5298857377
H,0,-2.2794889062,-1.0375805147,-0.5832622903
H,0,-0.1718826223,2.4486997375,0.7722826236
H,0,0.0963780365,1.9834601888,-0.8959426696
H,0,-1.7980536945,0.7273096305,1.1311553546
H,0,-2.1960614724,1.4175250097,-0.4320383626
O,0,1.5941324992,-0.1312406587,-0.4987569201
C,0,0.9365472419,-0.7280886117,0.6129232438
C,0,1.0560709105,0.7278247942,0.5094997254

H,0,-2.9417784178,4.0726147211,2.6835581183
H,0,-1.8940143539,4.9393988953,3.819119792
C,0,-2.2113159358,2.8902300117,4.3230410784
H,0,-1.2479012687,2.6959479937,4.7996692494
H,0,-2.9074823244,3.2416171777,5.0989744426
O,0,-3.7554737641,0.1967736229,1.508570886
O,0,-2.644749358,1.6620667409,3.7896182366
C,0,-4.3446342556,0.3376870552,2.7787826266
C,0,-4.0109879513,1.640129298,3.4653105364
H,0,-3.9663255943,-0.4915901426,3.380340047
H,0,-5.4374148627,0.2397059913,2.7193621
H,0,-4.2748038186,2.4782485272,2.8088821341
H,0,-4.6225370179,1.7268007254,4.3771277437
E = -614.884460
Nimag = 0

Propylene oxide

0 1
C,0,-0.0777395267,0.1327440075,-0.022028568
C,0,1.3809069985,0.1384373105,-0.0011568403
O,0,0.6481594397,1.3568682299,0.0205647396
H,0,-0.5864647742,-0.0988269002,0.9123782344
H,0,1.9214634478,-0.0590970413,-0.9233668091
H,0,1.9132145151,-0.1000725342,0.915449117
C,0,-0.8527069749,-0.1429426372,-1.2706334951
H,0,-1.1250335801,-1.1994079401,-1.324474218
H,0,-1.7738589271,0.4433590931,-1.2917836413
H,0,-0.2615317781,0.1099841819,-2.1520574593
E = -192.966995
Nimag = 0

1,4-dioxane

0 1
C,0,1.1606144223,-0.7529748143,-0.0650233161
C,0,1.1600901973,0.7537732254,0.0651010174
C,0,-1.1606426303,0.7529722382,0.0650384085
C,0,-1.1601185644,-0.7537724015,-0.0650931088
H,0,2.0208642923,-1.1850925566,0.4489899066
H,0,2.0200730036,1.1864850165,-0.4488597752
H,0,-1.2094500086,1.02993817,1.1282060623

H,0,1.8255481122,1.2168772862,1.1032131266
H,0,1.6076274693,-1.2518716785,1.291848005
E = -309.616611
Nimag = 0

Dibenzyl ether

0 1
C,0,-2.3600182622,0.360390228,-0.8121732705
C,0,-2.373707041,-0.3078561603,0.4066082547
C,0,-3.548386027,-0.9259722208,0.8253028101
C,0,-4.6928322214,-0.8710651281,0.0449274595
C,0,-4.6739190939,-0.2019243031,-1.1718009096
C,0,-3.5040390666,0.4098679354,-1.5975238443
H,0,-1.4466455728,0.8362616206,-1.1449033245
H,0,-3.5659078172,-1.459323225,1.7702580943
H,0,-5.5995810287,-1.3583131156,0.3836171916
H,0,-5.5656009691,-0.1624841404,-1.7859475156
H,0,-3.4793577374,0.930499425,-2.5477435393
C,0,-1.1637786472,-0.3454250625,1.2931261638
H,0,-1.2727681078,0.4027806305,2.0945032424
H,0,-1.0924053491,-1.3267421217,1.7845750779
O,0,-0.0024336511,-0.0839079103,0.5518039745
C,0,1.1491776273,-0.0450811271,1.351359282
H,0,1.2510560623,-0.9909523631,1.9071068867
H,0,1.068546638,0.7551991046,2.1013950144
C,0,2.3695045004,0.1721295622,0.5059187676
C,0,2.3764059198,-0.1326898034,-0.850358676
C,0,3.5329892486,0.6579040678,1.0952944539
C,0,3.5296280052,0.0447609422,-1.6029242001
H,0,1.4716248566,-0.5035660968,-1.3143358165
C,0,4.6867602293,0.8288174689,0.345968346
H,0,3.5342487757,0.909137674,2.1510201566
C,0,4.6883654237,0.5226548117,-1.0085164749
H,0,3.5210692068,-0.1925346245,-2.6603761312
H,0,5.5845597307,1.209407111,0.8186350681
H,0,5.5871963682,0.6608108205,-1.5974565418
E = -616.639258
Nimag = 0

2-chloroethyl ethyl ether

0 1
C,0,-5.3770272115,-2.0245051154,0.0035316333
H,0,-4.9634653589,-3.0323422836,-0.0559324807
H,0,-5.0460186028,-1.4638182004,-0.8720070001
H,0,-6.4655975067,-2.0953313938,-0.0231934578
C,0,-4.9294387731,-1.3433817491,1.2709903661
H,0,-5.3530960905,-0.3304528906,1.3381266543
H,0,-5.2753453484,-1.9000162221,2.1538307117

H,0,-1.2086751835,-1.0307688015,-1.1282647405
O,0,-0.0004481425,1.3066222898,-0.522738208
O,0,0.0004216768,-1.3066234715,0.5227490954
H,0,1.2094299476,-1.029942186,-1.1281908935
H,0,1.2086535146,1.030771321,1.1282725729
H,0,-2.0208911393,1.1850971234,-0.4489704334
H,0,-2.020099386,-1.1864911528,0.4488644124
E = -307.441657
Nimag = 0

Dimethoxymethane

0 1
O,0,-1.8950540351,0.2688202747,-0.0001949998
C,0,-2.4578339891,1.5564048037,0.0000094589
H,0,-2.1646661977,2.1251881013,-0.8917586036
H,0,-3.5396385731,1.4364316863,0.0020035943
H,0,-2.1615008456,2.1262873033,0.8900276206
C,0,-0.5091016482,0.3089297009,-0.0027190582
H,0,-0.1356862538,0.8387156206,-0.9008796357
H,0,-0.1324522078,0.839897267,0.893391096
O,0,-0.0849333399,-1.0111310511,-0.0026145869
C,0,1.3166040334,-1.1125316743,-0.0050932956
H,0,1.5640937416,-2.1724597206,-0.0048396344
H,0,1.7535067248,-0.6454512462,-0.8970931741
H,0,1.7567437021,-0.6442693795,0.8846928685
E = -269.353711
Nimag = 0

Epichlorhydrin

0 1
C,0,-0.0783728844,0.1359366649,-0.0073681107
C,0,1.3777347142,0.1302667597,-0.006283092
O,0,0.6488765049,1.3538551774,0.0003982143
H,0,-0.6028065213,-0.0920142218,0.917480063
H,0,1.9063107354,-0.0883335382,-0.9299401274
H,0,1.9108734168,-0.095226899,0.9118533393
C,0,-0.8436834427,-0.1279739664,-1.2652134783
H,0,-1.7851899575,0.4183942586,-1.2815857429
H,0,-0.2528045879,0.1388447419,-2.1394794342
Cl,0,-1.2453448774,-1.8645903671,-1.3808141209
E = -652.432059
Nimag = 0

Bis(2-chloroethyl)ether

0 1
C,0,-4.2476283671,0.2328863724,0.0258660868
H,0,-3.8786029317,-0.7906845508,0.0310690894
H,0,-3.875867136,0.7474361961,-0.8578401081
C,0,-3.8471239972,0.9606210239,1.2863310869
H,0,-4.2516966005,1.9811724731,1.2771602139
H,0,-4.2544262984,0.4442368826,2.1653880664
O,0,-2.4419136171,0.9783724239,1.3213643092
C,0,-1.9358622235,1.6339993952,2.4572445443
H,0,-2.275606191,1.1414764654,3.3777248106
H,0,-2.2728509928,2.6783616503,2.4894098237
C,0,-0.4301954308,1.5777648068,2.3646234164
H,0,-0.0739239891,2.0867934323,1.4713905024
O,0,-3.5208693325,-1.2746120325,1.273689369
C,0,-3.0093583497,-0.6570218596,2.4193613003
H,0,-3.3224077166,0.3986088022,2.4648906646
H,0,-3.3750632796,-1.1509013442,3.330389176
C,0,-1.5074770857,-0.6897960947,2.3819555181
H,0,-1.1365290008,-0.3150804496,1.4296521804
H,0,-1.0944214577,-0.0947839194,3.1940899207
Cl,0,-0.8661020555,-2.3482444371,2.5719311741
E = -692.945432
Nimag = 0

1,3-dioxane

0 1
C,0,-1.3162949174,0.0009981177,0.0480114553
C,0,1.443642124,-0.0009489321,0.2041219325
C,0,0.6340748789,1.2336797032,-0.1482299938
H,0,-2.2907085038,0.0016904978,0.5340403142
H,0,1.6582278178,-0.0010636813,1.2754093028
H,0,0.5285305873,1.3184279996,-1.2404938484
O,0,-0.652801941,1.1636681408,0.4440437775
H,0,-1.4351044481,0.0010502666,-1.0506098034
H,0,2.3943035701,-0.0016374634,-0.3348159626
H,0,1.1003574362,2.1473475766,0.22020571
O,0,-0.6544447323,-1.1626048111,0.4440967441
C,0,0.6323328522,-1.2344599873,-0.1481504611
H,0,0.526688485,-1.3191362486,-1.2404107175
H,0,1.0973387909,-2.1487551786,0.2203475505
E = -307.450688
Nimag = 0

1,3-dioxolane

0 1
C,0,-0.924141892,-0.7685805917,0.1886419238
O,0,0.3894905517,-1.1273524156,-0.1982603481
C,0,1.1574339569,-0.0261137324,0.1848393091
C,0,-0.9665574043,0.7390264198,-0.075500352
H,0,-1.6331651667,-1.3393192093,-0.4083730897
H,0,-1.0828320754,-0.9907795655,1.2509913072
H,0,1.3645536292,-0.0589522976,1.2674924449
H,0,2.0886394151,-0.0189574085,-0.3826702767
H,0,-1.485090305,1.2817139222,0.7191052178
H,0,-1.4308079545,0.9797134733,-1.0342985016
O,0,0.4037302451,1.1187944051,-0.1280426348
E = -268.161886
Nimag = 0

Cyneole

0 1
C,0,-0.8148733183,-0.5396321706,-0.0928380256
C,0,0.7130553521,-0.5264539368,-0.0565116344
C,0,-0.1201621664,1.8656678297,0.0035729372
C,0,-1.3163794715,0.9137740098,-0.0059039505
H,0,-1.1394163726,-1.0106030231,-1.0236849551
H,0,-1.2241462216,-1.1450077333,0.7163164239
H,0,-1.9728777815,1.162274165,-0.8431345621
H,0,-1.8865281796,1.08047242,0.9110935151
C,0,1.1891530864,0.1892341672,-1.3203082949
H,0,2.2725210665,0.1264591615,-1.4256084574
H,0,0.7657291931,-0.3173599924,-2.1907654917
C,0,0.7227627807,1.6553669766,-1.2543365119

H,0,-0.0766813489,0.5487292069,2.3603974284
Cl,0,0.290404497,2.3917204062,3.7750765303
Cl,0,-6.023228863,0.1673786057,-0.0929512807
E = -1152.410532
Nimag = 0

2,3-dihydrofuran

0 1
C,0,1.1882888843,-0.0911990371,-0.0620919685
C,0,0.5799951327,1.0879247108,-0.0442465274
C,0,-0.9009558309,0.8559436935,0.0853646088
C,0,-0.9791651233,-0.6602121517,-0.1394202854
O,0,0.3644102909,-1.1696791331,-0.0072411912
H,0,2.2431451525,-0.3252030564,-0.0976711954
H,0,1.069437535,2.0489279144,-0.0554810414
H,0,-1.4927335087,1.4010102081,-0.6524688728
H,0,-1.3219371438,-0.8966199964,-1.150576005
H,0,-1.6079246484,-1.1839830982,0.5791312062
H,0,-1.2666387403,1.1503219462,1.0749672721
E = -231.048973
Nimag = 0

Ethyl vinyl ether

0 1
C,0,-3.9377024914,-0.5312816978,0.027708362
H,0,-3.5572093042,-1.5536806127,0.0289219258
H,0,-3.5544655667,-0.0206825955,-0.8568765175
H,0,-5.0260907845,-0.5681036038,-0.0393840369
C,0,-3.5230095448,0.1920916587,1.2808776507

H,0,1.5739990581,2.3386915195,-1.2073193525
H,0,0.1374018882,1.9300568945,-2.1350184739
H,0,1.1050249784,-1.5462066158,-0.0220570244
C,0,1.20216296,0.2300049743,1.19123545
O,0,0.6861705736,1.5728770248,1.1468444549
C,0,-0.5488050569,3.3061277771,0.1590779717
H,0,0.3247457423,3.9593654252,0.1986455553
H,0,-1.1745295796,3.6115982957,-0.6813684398
H,0,-1.1175166742,3.4348533703,1.0817767289
C,0,2.721179477,0.3267185671,1.2450095239
H,0,3.0188092132,0.8703950712,2.1433907317
H,0,3.1750289459,-0.6665578294,1.2778411822
H,0,3.1181605316,0.864216713,0.3845427713
C,0,0.703305002,-0.4077831073,2.4812250886
H,0,1.0611043453,-1.4360322316,2.5725341567
H,0,1.0739281385,0.1621180688,3.3351003353
H,0,-0.3850133207,-0.410609601,2.5307851775
E = -466.774651
Nimag = 0

(Di-tret-buthyl ether)

0 1
C,0,-1.9992347551,-0.0290639999,-0.1186878733
C,0,-3.4569000879,-0.2974706923,-0.4916602625
H,0,-4.0600758877,0.5888551495,-0.2876477515
H,0,-3.530530932,-0.5247542201,-1.5574017491
H,0,-3.8791294815,-1.1385319109,0.055534395
C,0,-1.447265408,1.050302766,-1.0428857819
H,0,-2.0242140715,1.969653265,-0.9300945422
H,0,-0.4073118903,1.2642627136,-0.7913004447
H,0,-1.4986310487,0.7313223682,-2.0853628847
C,0,-1.1670462589,-1.2950404382,-0.2844779975
H,0,-0.1217341247,-1.1099789833,-0.0342762094
H,0,-1.5355796703,-2.1166355905,0.3295808872
H,0,-1.2151682385,-1.6180731182,-1.3264028801
O,0,-1.8950194772,0.58326171,1.1698254777
C,0,-1.9695418595,-0.0732414629,2.4385047665
C,0,-2.2064980263,1.0876496614,3.3976933591
H,0,-2.249855839,0.735079133,4.4296705873
H,0,-1.3977792425,1.8151315298,3.3108846812
H,0,-3.1458979787,1.587857017,3.1570593031
C,0,-3.1194781433,-1.064944529,2.5687640748
H,0,-4.0720411852,-0.591169782,2.3286933619
H,0,-2.9891485111,-1.9371308496,1.9282788206
H,0,-3.1670687167,-1.4205069413,3.6000769989
C,0,-0.644074619,-0.744493224,2.7969741168
H,0,0.1808885092,-0.0527644623,2.6182178925
H,0,-0.640954382,-1.0162253422,3.8548059073
H,0,-0.4673331136,-1.6518812666,2.2221281769
E = -390.604600
Nimag = 0

1,3,5-trioxane

0 1
C,0,-1.2892212378,0.2894954303,-0.0641397303
C,0,0.8954316947,0.9717960898,-0.0655678266
H,0,-2.2675621153,0.5093211877,0.3566174083
H,0,0.9218478864,1.0012163216,-1.1681170566
O,0,-0.3975933278,1.2741889908,0.3815395997

H,0,-3.9071394796,1.2188818408,1.2844299218
H,0,-3.9098690071,-0.316583067,2.171639394
O,0,-2.1015832252,0.2182670259,1.3305606085
C,0,-1.5694831123,0.8372020922,2.4032072551
H,0,-2.2867795489,1.2478984384,3.1116379665
C,0,-0.2603675664,0.9453357404,2.5943792821
H,0,0.4499860916,0.5356665686,1.887705359
H,0,0.1095593695,1.4525601617,3.4732086889
E = -232.249706
Nimag = 0

Ethyl ethynyl ether

0 1
C,0,-4.4899927973,-0.3902558014,0.0211181996
H,0,-4.0974198318,-1.4076302623,0.0295021903
H,0,-4.1019899533,0.1288658465,-0.8559249695
H,0,-5.5767332301,-0.4406551805,-0.060753848
C,0,-4.1176273576,0.3368079491,1.2808568125
H,0,-4.4883350987,1.3645676647,1.2867169028
H,0,-4.4835103557,-0.1751792746,2.1740271635
O,0,-2.6693738439,0.3817480723,1.3509520682
C,0,-2.1883779736,0.9781072938,2.3870844224
C,0,-1.709772149,1.5281551311,3.3419218273
H,0,-1.275189679,2.0116120015,4.1824853009
E = -230.984165
Nimag = 0

1,1,1,3,3,3-hexafluoroisopropyl methyl ether

0 1
C,0,0.0009394054,0.3721414903,-0.3134272995
H,0,-0.0030597259,0.592118702,-1.3864673046
C,0,-1.2894727101,-0.4030491487,-0.0324421407
C,0,1.2776541816,-0.4318581552,-0.0400304125
O,0,-0.0093217857,1.5111664431,0.4754088665
C,0,0.2716406379,2.719076579,-0.2131939473
H,0,0.207546481,3.5150045154,0.5253555821
H,0,1.2760250038,2.7068860159,-0.6436148917
H,0,-0.4661606219,2.8986507187,-1.000898131
F,0,1.360040605,-0.842330805,1.2224993675
F,0,1.3681890471,-1.5013346601,-0.8321359772
F,0,2.3393541805,0.3446135966,-0.2846487362
F,0,-1.3452244127,-1.5360826761,-0.7349790263
F,0,-1.4291966195,-0.7065573435,1.2547345986
F,0,-2.3336086665,0.3493857273,-0.3901585477
E = -828.665060
Nimag = 0

H,0,-1.328649092,0.2980370743,-1.1666528131
H,0,1.575285142,1.7093226704,0.3543976588
O,0,-0.9042142665,-0.9816678386,0.3821405096
C,0,0.3938294193,-1.261298165,-0.0651968566
H,0,0.4047702776,-1.2993974973,-1.1677451321
H,0,0.6930934121,-2.2186392869,0.3548698595
O,0,1.3027812074,-0.292508977,0.3803893793
E = -343.343657
Nimag = 0

Dichloromethyl methyl ether

0 1
C,0,-2.9499160926,0.0683893524,0.0065041606
H,0,-2.5534014074,-0.9389081214,0.0739522004
O,0,-2.5392741634,0.7631981542,1.0771400237
C,0,-2.9486832482,2.1218667154,1.1893945299
H,0,-4.0363235829,2.1919824768,1.2391914375
H,0,-2.5109921898,2.485892317,2.1146814895
H,0,-2.5761094695,2.7056863164,0.3463532165
Cl,0,-2.3570344242,0.7465401068,-1.5549098948
Cl,0,-4.7423238621,-0.0947309977,-0.0945871732
E = -1073.849035
Nimag = 0

Furan

0 1
C,0,-0.4100937671,0.1826904384,0.0002401235
C,0,0.9434003311,0.1679489266,-0.0001958015
C,0,1.3521813592,1.5366132742,0.0000673965
C,0,0.2122982164,2.2665638159,-0.0000498717
O,0,-0.8694582133,1.4547715381,-0.0008301763
H,0,-1.1583681846,-0.5918405512,0.0004197571
H,0,1.5743252982,-0.7059001852,-0.0001977445
H,0,2.3589999077,1.9213167065,0.0002560665
H,0,0.0113681824,3.3245993867,-0.0000697497
E = -229.850684
Nimag = 0

Nitriles

Optimized Cartesian coordinates, Electronic energies and the number of imaginary frequencies (all in atomic units). The computations are performed at: CPCM//PBE0-D3/Def2TZVPP.

Trichloroacetonitrile
0 1
Cl,0,0.5333456974,1.4604872532,-0.8242268722
Cl,0,0.5361002481,-0.0144150112,1.6759601475
Cl,0,0.5404184787,-1.4421638429,-0.8511603693
C,0,-0.0262919927,0.0000022268,-0.0000125152
C,0,-1.4839553501,-0.0034144412,-0.000514928
N,0,-2.6322660814,-0.0068291846,-0.0010014629
E = -1511.001053
Nimag = 0

Cyanogen bromide
0 1
C,0,0.,0.,-1.1287131294
N,0,0.,0.,-2.2815588366
Br,0,0.,0.,0.649524966
E = -2666.603331
Nimag = 0

Dibromoacetonitrile
0 1
Br,0,0.227663955,-0.4250330098,1.6135412553
Br,0,0.227663955,-0.4250330098,-1.6135412553
C,0,0.2220982305,0.6446414684,0.
C,0,-0.9238724441,1.5229779734,0.
N,0,-1.8273650287,2.23342561,0.
H,0,1.1445877468,1.2177669301,0.
E = -5279.144952
Nimag = 0

Chloroacetonitrile
0 1
C,0,1.2639723705,-0.1092095179,0.
N,0,2.2737399412,0.4384609757,0.
C,0,-0.0019992042,-0.8168524493,0.
H,0,-0.0759746819,-1.4425364616,-0.8881080637
H,0,-0.0759746819,-1.4425364616,0.8881080637
Cl,0,-1.3714987435,0.3165599146,0.
E = -592.100240
Nimag = 0

p-Trifluoromethylbenzonitrile
0 1
C,0,-2.1245969805,-0.0026288241,0.0027755326
C,0,-1.4271072757,-1.2072480327,-0.0868486671
C,0,-0.0451270561,-1.1961435332,-0.1018434859
C,0,0.6360859946,0.0114783889,-0.0295022653
C,0,-0.0551925837,1.2117262357,0.0608419729
C,0,-1.4375307967,1.2087481627,0.0762517765
C,0,-3.5522809735,-0.0096622381,0.0164918386
N,0,-4.7037121391,-0.0154120453,0.0278967605
C,0,2.1389419797,0.0127786958,0.0005304389
F,0,2.599443381,-0.1105924928,1.2559758808
F,0,2.6492447759,1.1460662976,-0.4932201349
F,0,2.6531519419,-1.0011361203,-0.7046969207
H,0,-1.9687156667,-2.1418436797,-0.1462479851
H,0,0.5026809897,-2.1261999218,-0.1761874927
H,0,0.484527021,2.147859957,0.1128341922
H,0,-1.9870756117,2.1382251503,0.1426865588
E = -661.106102
Nimag = 0

o-Fluorobenzonitrile
0 1
C,0,-0.498460471,-0.3717541056,-0.0000019725
C,0,0.0712022282,0.8992476618,0.0000183294
C,0,1.4378842697,1.0812643886,0.0000194149
C,0,2.2591300318,-0.0358457677,0.0000138079
C,0,1.71739381,-1.3153279787,0.0000065016
C,0,0.3458325048,-1.4848722642,-0.0000056035
C,0,-1.9135062858,-0.5250091151,-0.0000174151
N,0,-3.056753796,-0.6644357629,-0.0000159578
F,0,-0.7299272951,1.9614198277,0.0000038492

m-Trifluoromethylbenzonitrile
0 1
C,0,-1.8525508921,-0.0375340157,-0.0540003196
C,0,-0.5788277491,-0.6035375104,-0.0833840719
C,0,0.5265214768,0.2243620574,-0.0161055827
C,0,0.3757642321,1.6024992093,0.0821085341
C,0,-0.8929399588,2.1579340188,0.1114906271
C,0,-2.0110244358,1.3441745083,0.0439571036
C,0,-3.0017176607,-0.8812956462,-0.1272247195
N,0,-3.9304945696,-1.5594902422,-0.1858694892
C,0,1.9109117155,-0.3600878528,-0.0097934583
F,0,2.432144859,-0.3658852623,1.22763921
F,0,2.748392824,0.3490882412,-0.7779611462
F,0,1.9315424518,-1.6207816803,-0.4511118432
H,0,-0.4618568718,-1.675539158,-0.1627153188
H,0,1.2507307827,2.2384007417,0.1295964656
H,0,-1.0117359418,3.231026264,0.1851005325
H,0,-3.0056222622,1.7699213272,0.0643014766
E = -661.105985
Nimag = 0

α-Bromo-o-tolunitrile
0 1
C,0,-1.2260524536,0.8082989803,0.0827955141
C,0,-0.564692126,-0.3390275485,0.5447403721
C,0,-1.2286268487,-1.5567764833,0.4674848497
C,0,-2.5103893879,-1.6388824789,-0.0530664046
C,0,-3.1548406742,-0.4972256124,-0.5097816021
C,0,-2.5155874135,0.7275195593,-0.4416199004
C,0,-0.5814071912,2.0792818753,0.1495004863
N,0,-0.0615107706,3.1056050656,0.2105156654
C,0,0.8123307878,-0.2666102867,1.0977925946

H,0,1.8390167903,2.0860941271,0.00002546
H,0,3.3338459418,0.0967123486,0.0000205876
H,0,2.3661660853,-2.1812962362,0.0000043573
H,0,-0.091917814,-2.4743921234,-0.0000163589
E = -423.412150
Nimag = 0

o-Bromobenzonitrile

0 1
C,0,0.7530261985,0.8501698592,0.0000075969
C,0,0.1438190898,-0.4084437301,-0.000021843
C,0,0.9158507408,-1.5578286604,-0.0000287255
C,0,2.2987268167,-1.4560371052,-0.0000063552
C,0,2.9160428157,-0.212675712,0.0000229315
C,0,2.14752847,0.9357621252,0.0000299626
C,0,-0.0140611383,2.0510104207,0.0000153966
N,0,-0.6047781352,3.0396290731,0.0000224073
Br,0,-1.7308569317,-0.5587063599,-0.0000526897
H,0,0.4343367542,-2.5263219863,-0.0000516221
H,0,2.8955710959,-2.3596675302,-0.0000119848
H,0,3.9956362174,-0.1379853321,0.0000402892
H,0,2.6112790061,1.9136409381,0.0000526363
E = -2897.485516
Nimag = 0

Acrylonitrile

0 1
C,0,1.3143427664,-0.9714216274,0.
H,0,2.0145351519,-0.1457938463,0.
H,0,1.7165651534,-1.9759361558,0.
C,0,-0.0005247124,-0.7728439049,0.
H,0,-0.7015683781,-1.5998825889,0.
C,0,-0.5775080009,0.5288725313,0.
N,0,-1.0634529803,1.5735995919,0.
E = -170.690499
Nimag = 0

p-Fluorobenzonitrile

0 1
C,0,1.0548490163,-0.0000017316,0.0000326172
C,0,0.3591957093,1.210356018,0.0000320774
C,0,-1.0229997662,1.2130726023,0.0000251452
C,0,-1.6872852641,-0.000008886,0.0000227109
C,0,-1.0229894305,-1.2130858628,0.0000246379
C,0,0.3592055934,-1.21036317,0.0000315194
C,0,2.480592892,-0.0000074943,0.0000344272
N,0,3.6327503052,0.0001382975,0.0000440252
F,0,-3.0215979289,-0.0000140852,0.0000135283
H,0,0.9044391808,2.1449692177,0.000032951
H,0,-1.5869519733,2.1361548951,0.0000216327
H,0,-1.5869343631,-2.1361723962,0.0000207229
H,0,0.904456029,-2.1449724046,0.0000320045
E = -423.419792
Nimag = 0

Benzonitrile

0 1

Br,0,2.1487275434,-0.4515153033,-0.3294198704
H,0,-0.728563605,-2.4515354879,0.8191343262
H,0,-3.009398792,-2.5988149428,-0.1017310076
H,0,-4.1560579952,-0.5599655261,-0.9164607324
H,0,-3.0056437428,1.6276481023,-0.7891742292
H,0,1.0147532675,-1.0793284518,1.7888494972
H,0,1.0305194022,0.6879555389,1.5688804412
E = -2936.773183
Nimag = 0

o-Chlorobenzonitrile

0 1
C,0,2.3564798771,-0.5540397464,-0.0000014193
C,0,2.3142074159,0.8338434666,0.0000003606
C,0,1.0947624009,1.4838651188,0.0000008925
C,0,-0.0935793002,0.7501694048,-0.0000004547
C,0,-0.0375414137,-0.6474022001,-0.0000022226
C,0,1.1859333346,-1.2962271658,-0.0000027238
H,0,3.3093199004,-1.0686232903,-0.0000018728
H,0,3.2308581164,1.408965565,0.0000013701
H,0,1.0421462516,2.5647689924,0.0000022202
H,0,1.2129683189,-2.3775532831,-0.0000040774
Cl,0,-1.4873124459,-1.570220661,-0.0000038886
C,0,-1.3412225788,1.4377261403,0.0000000532
N,0,-2.3360178769,2.0179426588,0.0000007623
E = -783.691691
Nimag = 0

Methyl tiocyanate

0 1
C,0,-1.4636269947,0.8208338569,0.0000161306
S,0,-0.4620053607,-0.68787334,0.0001746149
C,0,1.073041163,-0.0069334592,-0.0002556584
N,0,2.1447495637,0.4271003412,0.0000368474
H,0,-1.2711994001,1.403567466,-0.8972734385
H,0,-1.2709182801,1.4039630601,0.8969874007
H,0,-2.4962736911,0.4738910751,0.0002591034
E = -530.705740
Nimag = 0

Phenyl cyanate

0 1
C,0,0.1338720428,-0.3672666234,-0.000035774
C,0,-0.0980645469,0.9926195553,-0.0000103805
C,0,-1.416527131,1.4291213631,0.0000147789
C,0,-2.4612919499,0.5173032247,0.000014831
C,0,-2.1915175671,-0.8451561674,-0.0000117689
C,0,-0.8827741853,-1.3012135961,-0.0000372317
O,0,1.4359147705,-0.901613754,-0.0000645242
C,0,2.4292203129,-0.097321365,-0.000066675
N,0,3.359825909,0.5855990025,-0.000032607
H,0,0.7258589972,1.6959597258,-0.0000110182
H,0,-1.6200673527,2.4925872546,0.0000348641
H,0,-3.4857739034,0.8671771874,0.0000347553
H,0,-3.0031804,-1.5618764314,-0.0000123195
H,0,-0.6453189961,-2.356909376,-0.0000579303
E = -399.377764
Nimag = 0

Benzyl cyanide

0 1

C,0,-0.6041708141,0.0000031775,0.0000057522
C,0,0.0890296638,-1.2111418055,0.0000040558
C,0,1.4729940799,-1.2041337133,0.0000098621
C,0,2.1643438993,-0.0000039399,0.000013178
C,0,1.4729998283,1.2041292977,0.000009755
C,0,0.0890355387,1.2111446688,0.0000041794
C,0,-2.0316610063,0.0000079345,0.0000063326
N,0,-3.1838846914,-0.0000035261,-0.0000029065
H,0,-0.4599072176,-2.1438343873,0.0000046284
H,0,2.0139842887,-2.1420886941,0.0000136603
H,0,3.2475395633,-0.000006543,0.0000172208
H,0,2.0139946586,2.1420816007,0.0000135585
H,0,-0.4598967911,2.1438399298,0.0000047233
E = -324.226848
Nimag = 0

o-Tolunitrile

0 1
C,0,0.4250945899,-0.4334878411,0.0000330275
C,0,-0.0065031051,0.9022349216,-0.0000088717
C,0,-1.3767026167,1.1318999139,-0.0000495858
C,0,-2.2854113859,0.0833603412,-0.0000481858
C,0,-1.841993771,-1.2317264195,-0.000008745
C,0,-0.4839275354,-1.4919968475,0.0000320869
C,0,1.8229923363,-0.7178363329,0.0000825756
N,0,2.9539504972,-0.9400039257,0.0000541801
C,0,0.9767400499,2.0282531561,0.0000057601
H,0,-1.7361938094,2.1543251257,-0.0000850477
H,0,-3.3477323023,0.2959031663,-0.0000812873
H,0,-2.5500214332,-2.0505537775,-0.0000110298
H,0,-0.1150422936,-2.5097063813,0.0000615688
H,0,1.6261352772,1.9816684544,0.8773421675
H,0,0.4681906043,2.991288777,-0.0002024125
H,0,1.6264408976,1.9814416693,-0.8770902007
E = -363.512913
Nimag = 0

Propionitrile

0 1
C,0,0.481046203,0.647906222,-0.0001031065
C,0,1.5388618503,-0.4525893342,0.0000140976
C,0,-0.8731240394,0.1174123847,-0.000429393
N,0,-1.9363236863,-0.3209536546,0.000481641
H,0,0.5877414063,1.290331657,0.8774264442
H,0,0.5879696978,1.2903891374,-0.8775533861
H,0,1.4457162419,-1.0837125413,-0.8836166624
H,0,1.4454770562,-1.0837765072,0.8835747884
H,0,2.5321732702,-0.0041523638,0.000166577
E = -171.927000
Nimag = 0

Trimethylsilyl cyanide

0 1

C,0,2.5054825135,0.0001965762,-0.4095690514
C,0,1.8441865992,-1.2003316669,-0.1920141214
C,0,0.526302883,-1.1999396917,0.2407039212
C,0,-0.139971325,-0.0001501606,0.4601659896
C,0,0.5261288085,1.1998161096,0.2411353271
C,0,1.8440119474,1.2005521435,-0.1915779787
H,0,3.535119683,0.0003325277,-0.746183644
H,0,2.3553743115,-2.1409682898,-0.3578378876
H,0,0.011845177,-2.139547857,0.4093144864
H,0,0.011532403,2.1392890608,0.4100759373
H,0,2.3550653176,2.1413212756,-0.3570637006
C,0,-1.5802849654,-0.0003271778,0.9189397677
H,0,-1.7915503492,-0.8794768516,1.5321394409
H,0,-1.7915920347,0.8783706572,1.5327669845
C,0,-2.5124343494,0.000206956,-0.2008656953
N,0,-3.23953362,-0.0000816114,-1.0915487754
E = -363.505988
Nimag = 0

Acetonitrile

0 1
C,0,0.,0.,0.277451
C,0,0.,0.,-1.170854
H,0,-0.0000003047,1.02463,-1.542916
H,0,0.8873557618,-0.5123147361,-1.542916
H,0,-0.8873554571,-0.5123152639,-1.542916
N,0,0.,0.,1.427024
E = -132.647419
Nimag = 0

Butyronitrile

0 1
C,0,-2.289734291,0.0171468476,0.0000324544
H,0,-2.477249334,0.6330386028,0.8828065332
H,0,-2.477351825,0.6330425179,-0.8827180439
H,0,-3.0166712856,-0.7961010651,0.000072072
C,0,-0.8722140224,-0.5249446298,-0.0000449505
H,0,-0.7070577321,-1.1558543576,0.8763557428
H,0,-0.7071419617,-1.1557925209,-0.8765031357
C,0,0.1560461905,0.6079716884,-0.0000748201
H,0,0.0220543447,1.2458279697,-0.8784004228
H,0,0.0221267161,1.2457861829,0.8782967715
C,0,1.5254424308,0.1220292422,-0.0001992883
N,0,2.6014927697,-0.2839644782,0.0002480876
E = -211.207950
Nimag = 0

Isobutyronitrile

0 1

C,0,-0.8386652713,0.6428725906,-1.6684565818
C,0,-0.8397001942,-1.7660833313,0.2774761389
C,0,-0.8396862739,1.1237006069,1.3907665248
Si,0,-0.3212573793,0.0000788275,0.0001899926
C,0,1.5578146075,-0.0002188293,0.0006766312
N,0,2.7105033128,-0.0003515225,0.0007394469
H,0,-0.4660095546,1.6556499266,-1.8319505527
H,0,-1.9291775985,0.6657992389,-1.7354337802
H,0,-0.4615865575,0.0044232144,-2.4694095196
H,0,-0.4665848565,-2.1394210544,1.2328401005
H,0,-0.4636402173,-2.4148340228,-0.5156298094
H,0,-1.9302647078,-1.8355879112,0.2865433721
H,0,-0.4687752353,0.7581530033,2.3499935093
H,0,-1.9302359932,1.1712054709,1.4424436584
H,0,-0.4614770808,2.1362727923,1.2392428692
E = -501.841293
Nimag = 0

o-Methoxybenzonitrile

0 1
C,0,0.2596462763,0.8551543617,-0.000014374
C,0,-0.3260269093,-0.4237867406,-0.000000508
C,0,0.5000209607,-1.5434362443,0.0000176611
C,0,1.8783058544,-1.38599959,0.0000225028
C,0,2.4593361426,-0.1260670625,0.0000090484
C,0,1.6461617023,0.9926646441,-0.00001011
C,0,-0.5722963848,2.0096727576,-0.0000315418
N,0,-1.2286982806,2.9568665096,-0.0000501671
O,0,-1.6645403233,-0.4616677586,-0.0000099988
C,0,-2.3032732305,-1.7236188721,0.0000390511
H,0,0.0746594083,-2.5367855411,0.0000266346
H,0,2.5056212632,-2.2693018352,0.0000365704
H,0,3.5355859243,-0.0159572844,0.000013063
H,0,2.0723580908,1.9876986639,-0.0000208391
H,0,-3.3704190334,-1.5173095431,0.0000525351
H,0,-2.044215616,-2.2977386721,0.8936256401
H,0,-2.0442488449,-2.2977917927,-0.8935231676
E = -438.675824
Nimag = 0

Trimethylacetonitrile

0 1
C,0,0.7648795453,1.3186160307,0.6094666659
C,0,0.7648290977,-1.1869822707,0.8372091931
C,0,0.7649860505,-0.1314415043,-1.446461212
C,0,0.2780612531,0.0000766697,0.0000507175
C,0,-1.1887152422,-0.0001078483,-0.0000832136
N,0,-2.3392470326,-0.0003138228,-0.0001227868
H,0,0.4154161902,2.173165177,0.0289029942
H,0,1.8564417565,1.325938004,0.6129827216
H,0,0.4152227311,1.4302216205,1.6364631178
H,0,0.4156075885,-2.1321534928,0.4198995904
H,0,0.4147542849,-1.1118305062,1.8673824216
H,0,1.8563937068,-1.1934440143,0.8424233332
H,0,0.4157007316,-1.0616619088,-1.8960597628
H,0,1.8565530855,-0.1318266693,-1.4545654107
H,0,0.4151392532,0.7019875356,-2.0567563695
E = -250.490435

C,0,-0.3549766383,0.0001453565,0.4000680049
H,0,-0.4338454818,0.0001077471,1.4921237084
C,0,-1.0129582211,-1.2681620083,-0.1426702365
H,0,-0.9409730352,-1.3026489379,-1.2308108922
H,0,-0.5453323357,-2.1653158341,0.2628369634
H,0,-2.0684453668,-1.272519031,0.1323045843
C,0,-1.0119511998,1.2690123429,-0.1425181684
H,0,-2.0674256238,1.2741141553,0.1324856708
H,0,-0.5436309239,2.1657528609,0.2631005235
H,0,-0.9399530941,1.3035747594,-1.230655837
C,0,1.0737617195,-0.0003155599,0.0970273126
N,0,2.1958742008,-0.001430851,-0.156195634
E = -211.208306
Nimag = 0

Hexanenitrile

0 1
C,0,-3.5254229556,0.1097844107,0.0000062983
H,0,-3.638936641,0.7457209374,-0.881710404
H,0,-3.6389362121,0.7456191203,0.8817964794
H,0,-4.3474231167,-0.6084251079,-0.0000348715
C,0,-2.17619795,-0.5882995645,-0.0000341519
H,0,-2.098413382,-1.2420598019,0.8752362473
H,0,-2.0984122906,-1.2419569532,-0.8753815148
C,0,-1.009681228,0.3862794989,0.0000260053
H,0,-1.0830870413,1.0406511624,0.8766655601
H,0,-1.0830865209,1.0407543539,-0.8765370183
C,0,0.3402053355,-0.3093493451,-0.000012125
H,0,0.4273094586,-0.9564805252,-0.8770810338
H,0,0.4273254199,-0.9565499503,0.8770053407
C,0,1.4929985105,0.6953227009,0.00000444
H,0,1.4354441693,1.3444219748,0.8783417066
H,0,1.4354223254,1.3444923163,-0.8782735891
C,0,2.7959610759,0.0521843084,-0.0001010434
N,0,3.8170250432,-0.4771795358,0.0003606744
E = -289.769102
Nimag = 0

p-Methoxybenzonitrile

0 1
C,0,-1.5325519199,-0.0248083741,-0.0000705355
C,0,-0.70360235,-1.1440804841,-0.0000885522
C,0,0.6726591089,-1.0013972073,-0.0000192626
C,0,1.2345152818,0.2752321535,0.000072628
C,0,0.4046266191,1.4002887292,0.0000880594
C,0,-0.9628236619,1.2537504421,0.0000170185
C,0,-2.9473083712,-0.1800113168,-0.000140384
N,0,-4.0935536839,-0.3049241565,-0.0001966525
O,0,2.5540650535,0.5210417859,0.0001309777
C,0,3.4433083048,-0.5784957532,0.0003425551
H,0,-1.1403125994,-2.1345701751,-0.0001615891
H,0,1.2949691459,-1.8847020454,-0.0000451475
H,0,0.8603413547,2.3820137759,0.000159475
H,0,-1.6025313183,2.1268337605,0.0000330148
H,0,4.4446660373,-0.1552012726,0.000556403
H,0,3.3115075131,-1.195408719,-0.8927999515

Nimag = 0

Cyclopropyl cyanide

0 1
C,0,-1.2537035398,0.7433846303,-0.2260006901
C,0,-1.2536044069,-0.7434119389,-0.225967858
C,0,-0.2142184019,0.0000726285,0.5791541131
H,0,-0.9132898453,1.253150601,-1.1166502262
H,0,-2.0127776374,1.2631953704,0.3421596142
H,0,-0.9131245963,-1.2531749319,-1.1165939869
H,0,-2.0126102562,-1.2632964081,0.3422164201
H,0,-0.3221780987,0.0000927771,1.6562666778
C,0,1.1432095032,0.000143031,0.1304525153
N,0,2.2350542792,-0.0002057594,-0.2360025793
E = 209.970647
Nimag = 0

p-(Dimethylamino)benzonitrile

0 1
C,0,-0.9182814403,0.0000154025,0.0001179396
C,0,-0.1860377088,-1.2068406819,0.0000096607
C,0,1.1899459187,-1.201873712,0.0000196692
C,0,1.903210541,-0.0000154387,0.0001227448
C,0,1.1899724908,1.2018565815,0.000214848
C,0,-0.1860135046,1.2068557663,0.0002047772
N,0,-2.2770850691,0.000015361,0.000158152
C,0,-3.0022927147,1.2468046228,-0.0005027058
C,0,-3.0022772394,-1.2467816581,-0.0001106472
C,0,3.3229933825,-0.0000387791,0.0001165083
N,0,4.4772697916,0.000022867,0.0001125653
H,0,-0.6998186223,-2.1571426894,-0.0001110135
H,0,1.727174912,-2.1420785059,-0.0000751297
H,0,1.7272193545,2.1420512237,0.0002971793
H,0,-0.6997673014,2.1571712525,0.0003038573
H,0,-4.0693143772,1.0395995738,-0.0008634317
H,0,-2.7749894039,1.8479373797,-0.8870970384
H,0,-2.7756977031,1.8484604511,0.8859150137
H,0,-4.0693017375,-1.0395915446,0.0003827287
H,0,-2.7750036685,-1.8484878832,0.8860904945
H,0,-2.7756339003,-1.8478575889,-0.8869211724
H,0,3.3111114857,-1.195382143,0.8934439432
E = -438.677928
Nimag = 0

1-Adamantanecarbonitrile

0 1
C,0,0.5126790475,1.2971472363,0.6504452299
H,0,0.8929917308,2.1596316381,0.0963848823
H,0,0.8931680116,1.369532127,1.6729067323
C,0,1.0304781082,-0.0000402831,0.0000742349
C,0,-1.0151542708,1.2891842717,0.6465497257
H,0,-1.371272484,2.213420636,1.1099700335
C,0,-1.5221045842,1.2043440861,-0.7931033177
H,0,-1.1786003898,2.0724241265,-1.3646847335
H,0,-2.6162207751,1.2219483108,-0.8046158637
C,0,0.5126247915,-0.0854284939,-1.4485190571
H,0,0.8928672541,-0.996413491,-1.9186453073
H,0,0.8930603713,0.7639086296,-2.0224294964
C,0,-1.0152109295,-0.0847747572,-1.439763293
H,0,-1.3712947322,-0.1453826049,-2.4719103521
C,0,-1.5224654672,-1.2890778624,-0.6466333128
H,0,-2.6165720079,-1.3077968089,-0.6561290571
H,0,-1.1789801575,-2.2182301647,-1.1124462473
C,0,0.5122200046,-1.2117329738,0.7982013302
H,0,0.8924119353,-1.1634629337,1.8222161702
H,0,0.8927164874,-2.1333744239,0.3495833732
C,0,-1.5224831981,0.0848103397,1.4395780724
H,0,-1.1789191955,0.1456857061,2.4771554101
H,0,-2.6165935041,0.0862643797,1.4606333384
C,0,-1.0156192004,-1.2043284066,0.7930785486
H,0,-1.3717650605,-2.0677871011,1.3617447934
C,0,2.4882577608,-0.0001320942,0.0000712418
N,0,3.6392974536,-0.0000840882,-0.0000760789
E = -482.588913
Nimag = 0

Dimethylcyanamide

0 1
N,0,-0.2939164005,0.0000002996,-0.2075768313
C,0,-1.0054357903,-1.2377649878,0.0688151697
C,0,-1.0055136071,1.2377164056,0.0688344364
C,0,1.0193696576,0.0000394394,-0.0578297209
N,0,2.1746778565,0.0000424764,0.0331871675
H,0,-1.2284564475,-1.3473749156,1.1355199627
H,0,-0.4056397694,-2.0845370117,-0.2587865585
H,0,-1.9415517506,-1.2343492536,-0.4903576419
H,0,-1.2285412561,1.3472954877,1.1355409326
H,0,-1.9416292111,1.2342497881,-0.4903386062
H,0,-0.4057712814,2.0845312723,-0.2587543101
E = -227.221875
Nimag = 0

E = -458.097463
Nimag = 0

Diethylcyanamide

0 1
N,0,-0.0083749238,0.3227265442,0.5318770917
C,0,1.2508083806,-0.4128126626,0.6869652579
H,0,1.1069985017,-1.0737146684,1.5438497028
H,0,1.4248642595,-1.0485233226,-0.1904979595
C,0,-1.2331335404,-0.4830473249,0.5686353781
H,0,-1.2877849583,-1.1239996781,-0.3204916441
H,0,-1.1335483024,-1.1382556987,1.4361019096
C,0,-2.4811183844,0.3607304238,0.6921744098
H,0,-2.6039638403,1.0220007281,-0.1668587703
H,0,-3.3557098211,-0.288753687,0.7429529838
H,0,-2.451567381,0.9719413103,1.5953129769
C,0,2.4322550267,0.4997157244,0.9239621108
H,0,2.5983658925,1.1677054165,0.0775019204
H,0,2.2833733383,1.1073986888,1.8176476454
H,0,3.3331736884,-0.0996181875,1.059815985
C,0,0.0009379186,1.3658202456,-0.2847493523
N,0,0.0068629354,2.3013189982,-0.9687778759
E = -305.781650
Nimag = 0

N1,N1-Dimethyl-N2-cyanoformamidine

0 1
N,0,1.2245637268,-0.0608157447,0.0001681359
C,0,2.2666866436,-1.0592956511,-0.0001484947
H,0,1.8304485866,-2.0565144188,0.0000946316
H,0,2.8955259654,-0.9495494854,-0.8869758527
H,0,2.8961655307,-0.9493890321,0.8861978319
C,0,-0.0543493688,-0.4046739351,0.0000959909
H,0,-0.2372754175,-1.4795840143,0.0001800068
C,0,1.6481879503,1.3209120862,-0.0000035111
H,0,2.251164799,1.5236047985,-0.8880758171
H,0,0.7746039858,1.966311949,0.0014488266
H,0,2.2536868992,1.5229093925,0.8864926866
N,0,-1.0194057263,0.4655154905,-0.0000289455
C,0,-2.2626596056,0.0353146795,0.0000838894
N,0,-3.3875379692,-0.2514931144,0.0001596214
E = -320.622215
Nimag = 0

trans-3-Dimethylaminoacrilonitrile

0 1
C,0,-0.0178845106,-0.3880096125,-0.0392193446
C,0,-1.0542897956,0.4907303214,0.0290873314
C,0,-2.3820641746,0.0284771352,-0.0153643141
N,0,-3.4804591929,-0.3344875859,-0.0479089336
N,0,1.2837780287,-0.0790105716,-0.0487489052
C,0,1.701996383,1.2964391168,0.0546862782
C,0,2.2943294432,-1.1028832584,0.0666658871
H,0,-0.2340820851,-1.4490802051,-0.1016857123
H,0,-0.9072614614,1.5583595616,0.1191731484
H,0,2.7765141382,1.3586570537,-0.105212295
H,0,1.4692765618,1.7156591305,1.0399302046
H,0,1.2038995934,1.9044958401,-0.704334417
H,0,3.0750187237,-0.9519557823,-0.6820919156
H,0,1.8461661146,-2.0818441108,-0.0950121664
H,0,2.7597012334,-1.0949910326,1.0577211542
E = -304.574007
Nimag = 0

N1,N1-Dimethyl-N2-cyanoacetamidine

0 1
N,0,-0.9138585793,0.3187388645,-0.6361079235
C,0,-0.5292654009,0.5078049356,-2.0223541499
C,0,-2.3185282524,0.4556862152,-0.3292196806
C,0,0.0264693196,0.0172671508,0.2608875794
C,0,-0.3548021431,-0.1827062478,1.694041004
N,0,1.2558869715,-0.0872451918,-0.180208087
C,0,2.2384077914,-0.3762690131,0.6385758599
N,0,3.1690268937,-0.628115444,1.2874991803
H,0,0.2101250283,1.304375071,-2.1119063971
H,0,-1.4161818503,0.7706175155,-2.5945683774
H,0,-0.0912410924,-0.4041777369,-2.4308069034
H,0,-2.5098976682,0.3023527172,0.7273174285
H,0,-2.9009737055,-0.2763085191,-0.89430657
H,0,-2.6664772743,1.4553080828,-0.6003702839
H,0,0.5320829893,-0.4117591284,2.2806469918
H,0,-1.0616624176,-1.0073873631,1.7984884129
H,0,-0.8195436099,0.7152910917,2.1040329161
E = -359.907984
Nimag = 0

Substituted Pyridines

Optimized Cartesian coordinates, Electronic energies and the number of imaginary frequencies (all in atomic units). The computations are performed at: CPCM//PBE0-D3/Def2TZVPP.

4-Pyrrolidinopyridine

0 1
C,0,-1.6837303437,-0.1176292249,0.4805191201
C,0,-0.8029614919,-0.9960445957,1.1385307115
C,0,0.5452601845,-0.9436786671,0.8476488473
N,0,1.1070272657,-0.1106464273,-0.0285987673
C,0,0.2680124343,0.7188764577,-0.6493114661
C,0,-1.0963096751,0.7644471623,-0.4453506433
H,0,-1.1646376625,-1.716546802,1.8593725785
H,0,1.2198670311,-1.6261640252,1.3578398912
C,0,-3.9639116426,0.705591666,0.0035715821
H,0,-3.7975017625,0.6461326903,-1.0752811351
H,0,0.7157000792,1.4041639338,-1.3642466199
H,0,-1.6927095104,1.4825565268,-0.9914023963
N,0,-3.0123659573,-0.1208692681,0.7229748177
H,0,-3.8697304337,1.7593566431,0.296560735
C,0,-5.3131834863,0.1308514408,0.4126996958
H,0,-6.1128433755,0.8691364836,0.3614824737
C,0,-5.0548070297,-0.3857619226,1.8234189024
H,0,-5.0730149202,0.4432356242,2.5352159112
H,0,-5.576718958,-0.6989516369,-0.247750576
C,0,-3.6448657476,-0.9527233644,1.7298911534
H,0,-3.1093043627,-0.8922163887,2.6810141956
H,0,-5.7810325173,-1.1283385708,2.1525394091
H,0,-3.6543381178,-2.0059777347,1.4204615793
E = -459.323153
Nimag = 0

4-N,N-Dimethylaminopyridine

0 1
C,0,-2.7791438549,-0.7128032632,-0.7553466512
C,0,-1.4093523534,-0.5495880853,-0.8116364742
C,0,-0.7953670877,0.4208099647,0.0020476813
C,0,-1.6603953191,1.1629242493,0.8276204476
C,0,-3.0163915419,0.9052593027,0.7934693805
N,0,-3.6016922969,-0.0124326841,0.0248068929
H,0,-3.2443222586,-1.464574919,-1.3873425148
H,0,-0.8331769129,-1.1718974192,-1.4814341857
H,0,-1.2882801229,1.9317932578,1.489618916
H,0,-3.6764934118,1.4834161916,1.4345502209
N,0,0.546310979,0.6278003051,-0.008768638
C,0,1.1273639908,1.6354235273,0.8432799735
H,0,2.2042969469,1.6521851449,0.6951286868
H,0,0.739202379,2.6335134707,0.6143153133
H,0,0.9351253943,1.431558158,1.9019269701
C,0,1.3903679106,-0.1586440076,-0.8735998318
H,0,2.4245378734,0.1498722033,-0.7419638821
H,0,1.3245864652,-1.2270703439,-0.6425345218
H,0,1.129030161,-0.0232271432,-1.9284294132
E = -381.954570

4-N,N-Diethylaminopyridine

0 1
C,0,-2.506249987,-0.784873911,-1.2444144455
C,0,-1.2146859357,-0.3011190931,-1.18761173
C,0,-0.8279331432,0.5360350746,-0.1229546972
C,0,-1.8303392552,0.788225209,0.8338574193
C,0,-3.0884710276,0.2452969728,0.6674146593
N,0,-3.4588552985,-0.5347081305,-0.3472041451
H,0,-2.7883629293,-1.4251434487,-2.076014153
H,0,-0.5232696147,-0.5815231547,-1.9688652799
H,0,-1.6422400944,1.3984379996,1.7052365848
H,0,-3.8509283162,0.4549592646,1.4129270668
N,0,0.4194186716,1.0749699761,-0.0334705445
C,0,0.7967673719,1.8861850857,1.1067592719
H,0,1.6473467371,2.4980701613,0.8013689876
H,0,-0.0119345197,2.5877641943,1.3281488635
C,0,1.4409573335,0.7462407198,-1.0076232043
H,0,1.0049939959,0.7887932954,-2.0093039991
H,0,2.1883748218,1.5408129617,-0.9735204475
C,0,2.1137737973,-0.6012579682,-0.7867741586
H,0,2.8315572145,-0.8009437972,-1.585078299
H,0,2.6490735819,-0.6217680739,0.1632843328
H,0,1.381446003,-1.4099230663,-0.7776918812
C,0,1.1570621737,1.092554443,2.3547883195
H,0,0.3352731363,0.4423300967,2.6584327104
H,0,2.0343493059,0.4677005267,2.1830324305
H,0,1.379285147,1.7693922023,3.1823626883
E = -460.519602
Nimag = 0

4-Piperidinopyridine

0 1
,0,1.0054901524,0.6339717199,-0.3860826114
C,0,1.9724127026,-0.1822226792,0.2325955902
C,0,3.2527841959,0.2966435326,0.4187320938
N,0,3.6783602567,1.5079528717,0.0588380171
C,0,2.7621577128,2.280713477,-0.5214912196
C,0,1.4522839253,1.9152537297,-0.7632552437
C,0,-0.6931248584,-1.1279356248,-0.319696638
N,0,-0.2720827223,0.2255636249,-0.6082162735
C,0,-1.2819019755,1.2070289624,-0.9878487225
C,0,-2.7096341519,0.7415030641,-0.7560623011
C,0,-3.0832710541,0.4916850501,0.7119888109
C,0,-1.963866276,-0.0479465204,1.598569819
C,0,-1.2679702214,-1.3040467705,1.092654756
H,0,1.7320239708,-1.1736644996,0.5895525008
H,0,3.986083496,-0.3436435437,0.9018115966
H,0,3.0936730429,3.2704159513,-0.8245738614
H,0,0.798873435,2.6171314974,-1.2609705488
H,0,-1.4355181103,-1.4152403674,-1.0631987196
H,0,0.148053815,-1.8010576591,-0.4832511992
H,0,-1.1625948624,1.4843556051,-2.0430660525

Nimag = 0

4-(4-Methylpiperidino)pyridine

0 1
C,0,1.5594932289,-0.1151740376,-0.2421093114
C,0,2.3371572888,-1.1694018678,0.2698616124
C,0,3.6658677062,-1.2791240519,-0.0846336482
N,0,4.3127024644,-0.4405565454,-0.8947717459
C,0,3.5811832553,0.5625031555,-1.3710143412
C,0,2.2441960669,0.7744442269,-1.0866557889
N,0,0.2193728087,0.0104563129,0.0407840442
C,0,-0.280493964,-0.4129972495,1.3401823843
C,0,-1.7579513579,-0.7521822597,1.2974581446
C,0,-2.5815649205,0.408321732,0.7608400688
C,0,-2.0166555865,0.805431671,-0.593752499
C,0,-4.0592723344,0.0703445767,0.6816048785
C,0,-0.5289934739,1.109376461,-0.5388131584
H,0,1.9156255657,-1.9227774036,0.919632915
H,0,4.2479306575,-2.1066297958,0.3115363424
H,0,4.0948478356,1.2633557219,-2.0236838568
H,0,1.7607595137,1.6414061358,-1.5117402169
H,0,-0.1128081748,0.3926684951,2.0732276624
H,0,0.2807676489,-1.2758811547,1.686132002
H,0,-1.9095222533,-1.6285931976,0.6572467067
H,0,-2.0869450774,-1.0249584689,2.3040746915
H,0,-2.4516694849,1.2578634816,1.445338476
H,0,-2.5380819877,1.6827201375,-0.9865509438
H,0,-2.1852667121,-0.0134111624,-1.302364805
H,0,-4.2255418637,-0.7738918713,0.0062285955
H,0,-4.6402123471,0.9162705417,0.3069092383
H,0,-4.4560647194,-0.2038569543,1.6618444785
H,0,-0.1742244412,1.2810262773,-1.5536068945
H,0,-0.3566863421,2.0368810936,0.0309629687
E = -537.885635
Nimag = 0

4-Aminopyridine

0 1
C,0,0.2009441211,1.1908920728,0.0116159779
C,0,0.9347348469,-0.0000003182,0.0190425367
C,0,0.2009430273,-1.190892037,0.0116162781
C,0,-1.1790116258,-1.1290879439,-0.0022859983
N,0,-1.8866163503,0.0000009739,-0.0094533587
C,0,-1.1790105888,1.1290892437,-0.0022862829
N,0,2.3017580773,-0.00000095,-0.013367646
H,0,0.7044099441,-2.1508018871,0.0153019739
H,0,-1.7513161675,-2.0526946435,-0.0065272806
H,0,2.7728524205,-0.8448381859,0.2588994255
H,0,-1.7513142821,2.0526964678,-0.0065277979

H,0,-1.1133656006,2.1179776847,-0.4024177803
H,0,-2.9128679225,-0.1450709992,-1.3589529322
H,0,-3.3622016514,1.5176740517,-1.1638950564
H,0,-3.9389985587,-0.1895412852,0.7426457632
H,0,-3.4297677343,1.4308837102,1.1531839159
H,0,-1.2079263622,0.7326654565,1.7293289552
H,0,-2.3676420618,-0.2398341322,2.5963263619
H,0,-1.9575484233,-2.1537067608,1.0874204929
H,0,-0.4649351587,-1.5581521478,1.7903284868
E = -537.875570
Nimag = 0

N-Methyl-N-pyridin-4-ylhydrazine

0 1
C,0,-0.1002528273,-0.1128581097,0.1195487435
C,0,0.8773428347,0.0177879159,1.1214975887
C,0,2.2049252808,-0.1789626396,0.7972545748
N,0,2.6564346613,-0.4953797547,-0.4159733466
C,0,1.7246937581,-0.6232734945,-1.3588426949
C,0,0.3683652728,-0.451835988,-1.1602594021
N,0,-1.4228210103,0.0866266351,0.3803767662
N,0,-2.3971446539,-0.2115253523,-0.5665315169
C,0,-1.8976883354,0.322964341,1.7212891595
H,0,0.6125602466,0.264637585,2.1396970281
H,0,2.9562190112,-0.0767196257,1.5755267417
H,0,2.0811904183,-0.8832404183,-2.3518509695
H,0,-0.3081547431,-0.5769681144,-1.9941241835
H,0,-2.2865918514,-1.1622796808,-0.9052611477
H,0,-2.3174012745,0.4169826083,-1.3574745196
H,0,-1.7123515175,-0.530575878,2.3844197056
H,0,-2.9678789224,0.4975634174,1.6640719121
H,0,-1.422863348,1.2061235532,2.1557455608
E = -397.966921
Nimag = 0

3,4-Dimethylpyridine

0 1
C,0,-0.4121204583,-0.4684192277,0.4925707097
C,0,0.2464467677,0.3363620549,-0.4446367806
C,0,1.6326590192,0.4116459934,-0.3688756711
N,0,2.3826314645,-0.2231608783,0.527038751
C,0,1.7455780131,-0.9832651094,1.4103705997
C,0,0.3677795904,-1.1330551419,1.4299105048
C,0,-1.9003116672,-0.6045051759,0.4811765586
C,0,-0.5035319729,1.092499938,-1.4940329282
H,0,2.1673045221,1.0304595809,-1.0860313923
H,0,2.3621759481,-1.4998618992,2.1402106977
H,0,-0.0962264124,-1.7679839375,2.1761650513

H,0,0.7044119196,2.1508014614,0.0153014318
H,0,2.7728531965,0.8448359217,0.2588992126
E = -303.415308
Nimag = 0

3,5-Dimethylpyridine

0 1
C,0,-0.7428491609,0.001553564,-0.0966938678
C,0,-0.1296983339,0.8611129561,-0.9991956621
C,0,1.1866342934,1.2236695785,-0.7277965524
N,0,1.87218093,0.799137569,0.3272563607
C,0,1.2585183643,-0.0216655537,1.171672583
C,0,-0.0533979297,-0.460732829,1.0169730747
C,0,-0.8469058705,1.3756543948,-2.2079598558
C,0,-0.6874092084,-1.3875075251,2.0066037022
H,0,-1.7697855954,-0.311628575,-0.2631595098
H,0,1.7097617759,1.8946640684,-1.4048208847
H,0,1.8396651743,-0.3558096798,2.0277531886
H,0,-0.2083551837,2.0357177943,-2.7957919932
H,0,-1.7416761303,1.9350932287,-1.9252335906
H,0,-1.168580901,0.5546001838,-2.8528860935
H,0,-1.002812841,-2.3173927614,1.5276792782
H,0,-1.5759370382,-0.9360911755,2.4541094898
H,0,0.0038376541,-1.6403112218,2.8111193072
E = -326.652885
Nimag = 0

4-tert-Butylpyridine

0 1
C,0,0.2076195028,0.2944714448,-0.021091559
C,0,1.0567151075,0.113850308,1.0708901607
C,0,2.4167082468,0.3249588578,0.9316533335
N,0,2.9969872647,0.7009719746,-0.2066853546
C,0,2.192021443,0.87381926,-1.2479129343
C,0,0.8165042694,0.6869185062,-1.207490198
C,0,-1.2903458475,0.0660742411,0.1169442682
C,0,-1.5374962141,-1.3843645313,0.5479676685
C,0,-2.0334574664,0.3178819849,-1.1915604288
C,0,-1.8448427615,1.0165373508,1.1845555352
H,0,0.6692609316,-0.1913031261,2.0351482172
H,0,3.0760389712,0.1837595198,1.7831561045
H,0,2.6654127632,1.180541435,-2.1764006843
H,0,0.2416819142,0.8520670327,-2.1084057535
H,0,-2.6097869942,-1.5622559165,0.6585650656
H,0,-1.060338648,-1.6090679995,1.5030916194
H,0,-1.150376967,-2.0835069454,-0.1963198232
H,0,-3.1006530286,0.1418559106,-1.0432845394
H,0,-1.6940210169,-0.3517014565,-1.9848045754
H,0,-1.9117261417,1.3477450353,-1.5341351106
H,0,-2.9197754226,0.8593682346,1.3007525799
H,0,-1.6807040538,2.0585431744,0.9018765918
H,0,-1.3752518517,0.8517807048,2.1555228165
E = -405.210723
Nimag = 0

4-Ethylpyridine

H,0,-2.2445963312,-1.265765237,1.275663367
H,0,-2.2497406173,-1.0043572455,-0.4744588854
H,0,-2.3835103812,0.3677362501,0.6095720784
H,0,0.1763669475,1.6604424742,-2.1293083764
H,0,-1.2147775076,1.7929581637,-1.047935847
H,0,-1.0813029247,0.4192833972,-2.1331464375
E = -326.654530
Nimag = 0

4-Methoxypyridine

0 1
C,0,0.1119350832,0.3948916343,0.0047606213
C,0,0.902864422,0.2225465669,-1.1268120616
C,0,2.2654914376,0.4629945906,-1.0135853556
N,0,2.8748015122,0.8475361814,0.1004695148
C,0,2.0983173644,1.0070750887,1.1743289803
C,0,0.7347244804,0.7987860038,1.185075614
O,0,-1.2147596397,0.201829034,0.0520367703
C,0,-1.8697086542,-0.2082116974,-1.1326511129
H,0,0.490893058,-0.0885601886,-2.076217168
H,0,2.8979262409,0.3332462877,-1.8874387109
H,0,2.598623571,1.3218566698,2.0853370839
H,0,0.1498158977,0.9425248142,2.0844038674
H,0,-2.9221309481,-0.3045953384,-0.8771635404
H,0,-1.4911880895,-1.1729557492,-1.4803873166
H,0,-1.7557297358,0.5356391023,-1.925510186
E = -362.529977
Nimag = 0

4-Methylpyridine

0 1
C,0,-0.4605870717,0.0804169136,0.1220609431
C,0,0.4233025922,-0.3209165656,1.1178195856
C,0,1.7874257067,-0.1803147061,0.9158770282
N,0,2.3242804718,0.3227353043,-0.19257676
C,0,1.4776292372,0.7033709439,-1.145587378
C,0,0.0992485109,0.6034233349,-1.0385017567
C,0,-1.942503957,-0.0228292118,0.3005021627
H,0,0.055118231,-0.7439951242,2.0455094867
H,0,2.4850843334,-0.4924926112,1.687578013
H,0,1.9235501085,1.1092411059,-2.048986387
H,0,-0.531471069,0.9292054802,-1.8577836344
H,0,-2.2054061553,-0.8516885941,0.9583890996
H,0,-2.4479854223,-0.1598050408,-0.6558798086
H,0,-2.3326905165,0.8933897712,0.7518354056
E = -287.369067
Nimag = 0

3-Ethylpyridine

0 1
C,0,0.0791981403,0.106918516,0.4026350033
C,0,0.764396787,1.1059143437,-0.2804980639
C,0,2.1430124296,1.1890618914,-0.1637851289
N,0,2.8743483533,0.3585580301,0.5747617184
C,0,2.2180259495,-0.5964519973,1.2283959883
C,0,0.8428438014,-0.7613202544,1.1754014325
C,0,-1.4052855822,-0.0557910639,0.2739426822
C,0,-1.7756122352,-0.9764604124,-0.8868843913
H,0,0.2293891921,1.8193769683,-0.8972880729
H,0,2.686581192,1.967981865,-0.6908282004
H,0,2.822511922,-1.2675108165,1.8319143748
H,0,0.3713541774,-1.5597515352,1.7374485764
H,0,-1.8662441437,0.9238824145,0.1248837162
H,0,-1.8080291196,-0.4617812197,1.2053025218
H,0,-2.8587324995,-1.084222272,-0.9667308567
H,0,-1.343477386,-1.9695069643,-0.7484504954
H,0,-1.4018949785,-0.5790254934,-1.8326258045
E = -326.649280
Nimag = 0

3-Methylpyridine

0 1
C,0,-0.8923858999,0.7321871595,-0.3676848007
C,0,-0.0632174069,0.1440016967,0.5823171183
C,0,1.2783273304,0.0016022081,0.2402655111
N,0,1.8071807145,0.3861282405,-0.9178140647
C,0,0.9944770892,0.9448081834,-1.8075547438
C,0,-0.3606660235,1.13885081,-1.5785874127
C,0,-0.5830250492,-0.3148126516,1.9084012687
H,0,-1.9478814655,0.8694797182,-0.1572256998
H,0,1.9643224318,-0.4527637296,0.9512785852
H,0,1.443367792,1.2525482861,-2.7472170541
H,0,-0.9820398195,1.5994942263,-2.336630761
H,0,-1.0208058588,0.51575879,2.4669142922
H,0,0.2114887102,-0.7485305515,2.5162908772
H,0,-1.3635955447,-1.068955386,1.7842568842
E = -287.367969
Nimag = 0

4-Vinylpyridine

0 1
C,0,0.265257904,-0.1881740309,-0.0468304013
C,0,1.0254045111,0.2462682911,-1.1337929364
C,0,2.3885494658,0.4202708159,-0.9800771841
N,0,3.0461517047,0.1981806633,0.1576980132
C,0,2.3234679184,-0.2154633033,1.193219781
C,0,0.9534493598,-0.4202260605,1.1425873961
C,0,-1.1820568622,-0.4050025591,-0.0985359137
C,0,-1.970994619,-0.227363267,-1.1548922651
H,0,0.5696326524,0.4496729927,-2.0943265467
H,0,2.9875879776,0.7580053124,-1.8205712822

0 1
C,0,-0.7999306714,0.7869513655,-0.6508816159
C,0,-0.1327159891,1.4020662629,-1.6963272339
C,0,1.2064770527,1.7239473057,-1.5301506671
N,0,1.8805775626,1.4736140632,-0.4121892305
C,0,1.222890721,0.883398369,0.5806157682
C,0,-0.1177496807,0.5108001078,0.5287596939
C,0,-0.7755991224,-0.1908220224,1.6791269433
C,0,-0.8132454345,-1.7045155248,1.4881142199
H,0,-1.8485645027,0.5245159156,-0.745645783
H,0,-0.6386674641,1.6335711147,-2.6253512345
H,0,1.7592410206,2.2071990833,-2.3300106279
H,0,1.7939303884,0.6937867031,1.4867080022
H,0,-1.7948089892,0.1870733302,1.7986304175
H,0,-0.2418059576,0.0486647434,2.6024292761
H,0,-1.3024604071,-2.1933023533,2.3326340438
H,0,0.1966328703,-2.1098227108,1.3980054699
H,0,-1.359294397,-1.969302753,0.580156558
E = -326.648226
Nimag = 0

4-Phenylpyridine

0 1
C,0,1.2249740103,0.3533038241,-0.0451695691
C,0,1.8398687367,1.4157627035,-0.7052031743
C,0,3.2111381911,1.5796533584,-0.5998612431
N,0,4.0027293714,0.775479973,0.1046821489
C,0,3.4154305152,-0.2382635184,0.7348384858
C,0,2.0540526683,-0.4896932801,0.692762146
C,0,-0.2302437046,0.1321330834,-0.1236742775
C,0,-0.7536296331,-1.1609996909,-0.1609265316
C,0,-2.1214835722,-1.3688649743,-0.2376582471
C,0,-2.9909126098,-0.2874461866,-0.2726042315
C,0,-2.4822742012,1.003417686,-0.2332075371
C,0,-1.114119241,1.2113837305,-0.1623396333
H,0,1.2617480765,2.0982896029,-1.3155868762
H,0,3.6981613329,2.4013575036,-1.1166351277
H,0,4.0668589303,-0.8869405134,1.3130284778
H,0,1.6432635759,-1.3225192723,1.2497970802
H,0,-0.0813834831,-2.0108289814,-0.1540305451
H,0,-2.5096854101,-2.3796367489,-0.2765555938
H,0,-4.0604210494,-0.4499950419,-0.3303010608
H,0,-3.154339268,1.8530451766,-0.2515084662
H,0,-0.7270046187,2.2222195867,-0.1120862273
E = -478.953211
Nimag = 0

Pyridine

0 1
C,0,-1.1216934118,-1.0556418154,0.0000231131
C,0,0.2656237903,-1.0600229973,-0.0000670811
C,0,0.9297292682,0.1563665839,-0.0003025386
C,0,0.1787614903,1.3211264309,-0.0004061391
C,0,-1.2045474123,1.2156579299,-0.0003063928
N,0,-1.8550337997,0.0547653798,-0.0001038503
H,0,2.0124505623,0.195833833,-0.0003558165
H,0,-1.6711074854,-1.9922797872,0.0001825562
H,0,0.8072776988,-1.9973134672,0.0000559241
H,0,0.6506337716,2.2954154413,-0.0005376962

H,0,2.8647758762,-0.3947536801,2.117536096
H,0,0.4238573807,-0.7590571947,2.025533368
H,0,-1.6266617522,-0.7442648715,0.8331923423
H,0,-3.0348235335,-0.4165781778,-1.0891951213
H,0,-1.5955169838,0.1087770696,-2.1142323458
E = -325.417983
Nimag = 0

4-Chloropyridine

0 1
C,0,-1.3535200869,-0.1096673747,0.2129971052
C,0,-0.489818316,-0.5069870592,1.2191218359
C,0,0.8704061755,-0.353160988,0.9948986175
N,0,1.3885979871,0.1496688523,-0.1212300708
C,0,0.5358433334,0.5228407585,-1.0702590902
C,0,-0.8429463305,0.4176247581,-0.9606332031
H,0,-0.8606824274,-0.9221548327,2.1462280877
H,0,1.5760389265,-0.6545172242,1.7629483562
H,0,0.9701140364,0.9320046341,-1.9772472053
H,0,-1.4940201367,0.7361433092,-1.7631787177
Cl,0,-3.0572123486,-0.2707936746,0.4206544688
E = -707.551430
Nimag = 0

3-Benzoylpyridine

0 1
C,0,-0.2343657302,-0.0409196206,-1.6901844045
C,0,0.6735893074,0.4763705197,-2.5953438779
C,0,1.7108911756,-0.3347824857,-3.0331497542
N,0,1.8749336199,-1.5965743227,-2.6429932328
C,0,0.9875203217,-2.09193271,-1.7920228852
C,0,-0.0771867819,-1.3577573203,-1.2740842714
C,0,-1.1126679416,-0.2271678852,1.1817113331
C,0,-1.9201366868,0.8834996461,1.3644743275
C,0,-1.4066647724,1.9911037728,2.0214042576
C,0,-0.099305466,1.986187881,2.4891743597
C,0,0.6914830098,0.8600581548,2.3099021126
C,0,0.1870032471,-0.2580190218,1.6624033056
C,0,-1.0275573634,-2.0566952557,-0.3718449576
O,0,-1.2873082489,-3.2210983607,-0.4779238817
O,0,-1.6791865509,-1.3276881341,0.5715237626
H,0,-1.0540249126,0.5637868824,-1.3250192199
H,0,0.5837525373,1.4923027123,-2.9569491704
H,0,2.4440591994,0.048393532,-3.7360322132
H,0,1.1162546101,-3.1254905926,-1.4878983297
H,0,-2.9360382747,0.8672557928,0.9902412249
H,0,-2.0331007318,2.8628644508,2.1658271841
H,0,0.2991929061,2.8543180591,2.9989979232
H,0,1.7082990563,0.8437159379,2.6826519362
H,0,0.7937564704,-1.1455686324,1.5334334717
E = -667.387491
Nimag = 0

3-Fluoropyridine

0 1

H,0,-1.8207580823,2.1097646082,-0.0003710787
E = 248.082955
Nimag = 0

4-Acetylpyridine

0 1
C,0,0.2564178606,-0.1804629009,-0.0160974335
C,0,0.9741282233,0.3009988567,-1.1044094902
C,0,2.3215511475,0.5899558092,-0.9395538989
N,0,2.9722560609,0.4321506612,0.2077510556
C,0,2.2784255441,-0.0295543988,1.2464327376
C,0,0.9324004324,-0.3475351753,1.1870401674
C,0,-1.2020706456,-0.523294999,-0.0866164563
O,0,-1.7744947364,-0.9389403182,0.8950193243
C,0,-1.9146347403,-0.3363467607,-1.3937671093
H,0,0.509820407,0.4544271837,-2.0696618531
H,0,2.902703393,0.9671638868,-1.7753845252
H,0,2.8279162256,-0.1514953336,2.1746389017
H,0,0.4035431836,-0.7205270536,2.0543986496
H,0,-2.956050825,-0.6268584127,-1.2799663126
H,0,-1.4468868464,-0.9415621394,-2.1738532472
H,0,-1.8564176433,0.7062804607,-1.7149327614
E = -400.622629
Nimag = 0

Methyl nicotinate

0 1
C,0,-4.4451788988,0.1727176337,0.5513800355
C,0,-3.1076835061,-0.0056187999,0.2060663778
C,0,-2.396519487,1.0894063294,-0.2697275666
C,0,-3.045631058,2.3040373572,-0.3792191086
C,0,-4.3817357701,2.3742296109,-0.0078348818
N,0,-5.076805489,1.3359387063,0.4493586395
H,0,-1.3556761128,0.968819234,-0.5429119681
H,0,-5.0240201938,-0.6640013249,0.9252042945
H,0,-2.5330672657,3.1844437865,-0.7445227364
H,0,-4.919878055,3.3142689399,-0.08221542
C,0,-2.4144848542,-1.3115595397,0.3250553385
O,0,-3.2118595322,-2.2720518674,0.7929138405
C,0,-2.6104451849,-3.5577460332,0.9339364684
H,0,-1.7719671295,-3.512324483,1.6288179665
H,0,-3.3900657892,-4.2081874016,1.3205040984
H,0,-2.2545621098,-3.9200059692,-0.0304689277
O,0,-1.2578393738,-1.4885863189,0.0320056097
E = -475.814428
Nimag = 0

3-Chloropyridine

0 1

C,0,-2.552519591,-0.5780538377,0.0000235396
C,0,-1.1686968099,-0.5536580369,-0.0000568779
C,0,-0.4837556126,0.642943723,-0.0002657801
C,0,-1.2427602247,1.802172742,-0.0003974716
C,0,-2.626669967,1.6985797273,-0.0002904816
N,0,-3.273073227,0.5366440756,-0.0000896296
H,0,0.5986166104,0.6567681917,-0.000323146
H,0,-3.0775679507,-1.5278195821,0.0001895248
H,0,-0.7663573271,2.774246122,-0.0005803208
H,0,-3.2425600058,2.5917307174,-0.0003976545
F,0,-0.4947529944,-1.7084161623,0.0000600578
E = -347.273866
Nimag = 0

3-Bromopyridine

0 1
C,0,-2.4598512544,-1.1595276638,0.0000367871
C,0,-1.0714317569,-1.1465711719,-0.0000345515
C,0,-0.3961672845,0.0614312295,-0.0002463413
C,0,-1.152891765,1.2216265291,-0.0004022369
C,0,-2.5359718281,1.1174633077,-0.0003134062
N,0,-3.1805439138,-0.0453696356,-0.0000928216
H,0,0.6855627109,0.0932737525,-0.0002997994
H,0,-2.9993348332,-2.1007024955,0.0002117936
H,0,-0.674944797,2.1929907195,-0.0005937098
H,0,-3.1536822112,2.0096498716,-0.0004288972
Br,0,-0.1223865368,-2.7770804733,0.0001696532
E = -2821.344420
Nimag = 0

C,0,-2.4596692035,-1.1587335375,0.0000311332
C,0,-1.0707263447,-1.1475389251,-0.0000400394
C,0,-0.3961755136,0.0611417433,-0.0002522196
C,0,-1.1529230611,1.2208171125,-0.0004003111
C,0,-2.5361880806,1.1172505664,-0.0003017504
N,0,-3.1807863563,-0.0455530916,-0.0000924617
H,0,0.6856969394,0.0882812941,-0.0003046626
H,0,-2.9951775856,-2.1022681761,0.00020676
H,0,-0.6752946144,2.1922938987,-0.0005913045
H,0,-3.1533631223,2.0097116468,-0.0004223854
Cl,0,-0.2028590573,-2.6387931215,0.0001376316
E = -707.550155
Nimag = 0

4-Cyanopyridine

0 1
C,0,-0.2477183421,-0.1490093454,-0.0650803613
C,0,0.4167402635,0.5621498188,-1.0596724837
C,0,1.7955994717,0.6659175358,-0.9716651201
N,0,2.5148830301,0.1254911478,0.007102822
C,0,1.8662803245,-0.5508618654,0.950100336
C,0,0.4912531417,-0.7206886503,0.9661862911
C,0,-1.6690347374,-0.2901767858,-0.1022052737
N,0,-2.814089552,-0.4037206729,-0.1324000041
H,0,-0.125208105,1.0197957564,-1.876085918
H,0,2.3457070215,1.2133169985,-1.7299793631
H,0,2.4732994515,-0.9832245954,1.7387042878
H,0,0.0084110319,-1.2801103423,1.755863787
E = -340.253307
Nimag = 0

3-Cyanopyridine

0 1
C,0,-0.8424979325,0.3059239046,-0.4719532817
C,0,-0.1617750889,0.9495384994,-1.486680608
C,0,1.2264632763,0.9354655353,-1.4705624007
N,0,1.9475888352,0.3338030905,-0.527864994
C,0,1.2974232407,-0.2835909698,0.4454970079
C,0,-0.0967962776,-0.3272780467,0.5202271376
C,0,-0.733140009,-1.0102696241,1.596671739
N,0,-1.2429443507,-1.5636158945,2.4687657456
H,0,-1.9242041708,0.2883518612,-0.4396435209
H,0,-0.6942755688,1.456074249,-2.2806557458
H,0,1.7860279714,1.4334489012,-2.2558266766
H,0,1.8952030744,-0.770717506,1.2087035978
E = -340.255013
Nimag = 0

3,5-Dichloropyridine

0 1
C,0,-1.3712668114,0.1285153901,0.3099092285
C,0,-0.4883699117,-0.332822158,1.2704373771
C,0,0.8758140225,-0.3380701896,1.0095422221
N,0,1.3753590029,0.0882082913,-0.1420262394
C,0,0.5456376859,0.5339938494,-1.0748740169
C,0,-0.8297946198,0.5689500537,-0.8849873317
Cl,0,-1.0756260998,-0.8998398439,2.7867045317
H,0,1.5734145921,-0.698095738,1.7574084828
H,0,0.9768879361,0.8774533747,-2.0084840965
Cl,0,-1.8553739496,1.1596361453,-2.1358756364
H,0,-2.4380818481,0.1441708305,0.4854454744
E = -1167.016054
Nimag = 0
